# Bayesian Estimation of the Variation in Strength and Aerobic Physical Performances in Young Eumenorrheic Female College Students during a Menstrual Cycle

**DOI:** 10.3390/sports9090130

**Published:** 2021-09-17

**Authors:** Shaher A. I. Shalfawi, Ghazi M. K. El Kailani

**Affiliations:** 1 Department of Education and Sports Science, University of Stavanger, 4036 Stavanger, Norway; 2 School of Sports Science, The University of Jordan, Amman 11942, Jordan; g.kailani@ju.edu.jo

**Keywords:** 1RM, aerobic, strength training, Bayesian Estimation, HDI + ROPE

## Abstract

Background: The purpose of the present investigation was to examine changes in strength and aerobic physical performances in young eumenorrheic female college students during the menstruation phase and different testing occasions within a menstrual cycle. Methods: A repeated measure experimental design used to investigate the variation in physical performance from different testing occasions compared to the menstruation phase. Twelve eumenorrhea female college students volunteered to participate in this study. The participants were 19.8 ± 0.8 (±SD) years old, with the body mass of 61.4 ± 11.6 kg, the height of 162.6 ± 5.1 cm, and BMI of 23.2 ± 3.8. All participants reported regular monthly menstrual cycles of 26–33 days, none of whom reported taking oral contraceptives in their entire life. None of the participants was an athlete, and their level of activity was limited to physical education classes and recreational activities. The menstrual cycles during the two cycles before testing had to be between 26 and 35 days to participate in this study. Second, there had to be no current or ongoing neuromuscular diseases or musculoskeletal injuries. Third, no one should be taking any dietary or performance-enhancing supplements that could have affected testing results during this study. The participants tested on one-repetition maximum (1RM) bench press, 1RM leg press, push-up to failure, leg press with 60% of 1RM to failure, and running 1600 m time trial. The participants were tested on four occasions based on the classical model of the menstrual cycle (i.e., 28 days; early follicular phase (menstruation phase) on day 2 (T1), late follicular phase on day 8 (T2), ovulation phase on day 14 (T3), and mid-luteal phase on day 21 (T4)). Data were analyzed using the Bayesian hierarchical model (Bayesian Estimation) with Markov Chain Monte Carlo simulation using the decision-theoretic properties of the high-density interval (HDI) + ROPE decision rule. Results: The Bayesian estimated difference from the four testing occasions neither showed that the most credible parameter values (95% HDI) were sufficiently away from the null value nor showed that the most credible parameter values are close to the null value (Rope odds ratio among all tests were spread in 12.7% < 0 < 87.3% with an effect size ranging between *d* = −0.01 and 0.44). Hence, no decision can be made as to whether strength and aerobic physical performances change during the menstruation phase compared to the other testing occasions within a menstrual cycle. Conclusions: It was noticed that different studies concluded different results, which make the research in menstrual cycle difficult. However, the results from this study and published studies suggest that future research should investigate and profile motivation and autonomic nervous system activity during the menstruation phase and examine the interaction effect of the three on performance compared to other testing occasions within a menstrual cycle.

## 1. Introduction

Studies of the key factors affecting female physical performance have been notably increased with the increased number of female participants in professional sports. Among these factors are the fluctuations in endogenous sex hormones during the menstrual cycle [1,2]. Females between the age of 13 and 50 experience fluctuations in endogenous sex hormones: among others, estrogen and progesterone [1,2]. Hence, females experience a different hormonal profile during the cycle length, which has been reported to be between 26 and 35 days [3]. Besides their primary role in reproduction, estrogen and progesterone influence other physiological systems. For instance, estrogen has a great role in developing connective tissues such as bone regeneration [4], muscles, tendons, and ligaments [5].

Furthermore, estrogen has been reported to greatly influence connective tissue adaptations to strength training [6]. Hence, estrogen can be seen as a metabolism regulator and anabolic hormone due to its receptors in muscles, bones, ligaments, and tendons [4,5,7]. Furthermore, studies have shown that a reduction in estrogen hormone is related to a reduction in muscle performance-related organs such as mitochondrial function, membrane microviscosity, and others [7,8]. Therefore, it was hypothesized that a low concentration of estrogen might be directly related to a higher injury rate [9] and performance reduction in the early menstrual bleeding phase [8]. By contrast, performance enhancement was expected when estrogen blood concentration was highest on the late follicular phase (i.e., between days 10 and 14 from the menstruation phase) [7].

Several authors have attempted to investigate the effect of different menstrual phases on physical performance [2,10,11,12,13,14,15,16,17,18,19,20,21,22,23,24,25,26,27,28,29,30]. The results of these investigations reported changes in performance on different testing days of the menstrual cycle [16,18,19,20,22,27,31] while others did not [12,13,14,15,17,21,23,24,25,26,28,32,33]. E.g., Romero-Moraleda et al. [28] investigated the effect of menstrual cycle phases on strength and power on 13 female eumenorrheic triathletes using no contraception. Force, velocity, and power output from half squat using 20, 40, 60, and 80% of one-repetition maximum (1RM) were assessed. The measures were obtained on three different occasions (i.e., day 3, day 13, and day 21 from the onset of menses) within a menstrual cycle. The authors reported no differences in the measured variables between the three test occasions. Tounsi et al. [25] investigated the effect of menstrual cycle phases on aerobic and anaerobic soccer-specific performance tests using 11 eumenorrheic female Tunisian high-level soccer players. The tests conducted were the five-jump test, the repeated shuttle-sprint ability test, and the Yo-Yo intermittent recovery test level 1. The measures were collected on three different occasions: day 2 to day 4 (T1), day 7 to day 9 (T2), and day 20 to day 22 (T3) from the onset of menses. The authors reported no differences in performance between the testing occasions. Gordon et al. [23] investigated the influence of menstrual cycle phases on endurance performance using 16 physically active female participants, where 10 of the participants were eumenorrheic (group 1) and 6 participants were using an oral contraceptive (group 2). The data were collected on four occasions (i.e., menstruation, mid-follicular, mid-luteal, and pre-menstruation). The authors reported no differences in VO_2max_, cardiac output, stroke volume, and heart rate from the different testing occasions for the 10 eumenorrheic participants, nor were there any differences observed between the two groups. Nevertheless, it seems that, irrespective of the participants’ background, the results were similar (e.g., while Romero-Moraleda et al. [28] and Tounsi et al. [25] used athletes, the results were similar to those by Gordon et al. [23], who did not use athletes).

Contrary to these studies, Rodrigues et al. [27] investigated the effect of menstrual cycle phases on strength performance using 12 healthy eumenorrheic females. The participants were tested on three different occasions: pre-menstruation (i.e., 2–3 days before the onset of menses), during the menstruation phase (i.e., day 1–2 from the onset of menses), and post menstruation (i.e., 2–3 days after the menstrual flow has stopped). The authors reported higher values in maximal voluntary contraction using leg press at 60% of 1RM from the test conducted on the post menstruation compared with the menstruation phase and the premenstrual period. Pallavi, UJ and Shivaprakash [22] investigated 100 healthy eumenorrheic females between 18 and 24 years, with regular menstrual cycles between 26 and 32 days. The authors assessed muscle strength (using a handgrip dynamometer) and fatigue rate (using Mosso’s ergograph) on three different occasions: menstruation, follicular, and luteal. The authors reported higher strength values and a lower fatigue rate during the follicular phase than the other two test occasions. Similar results were reported in a study by Ansdell et al. [26]. Finally, Bandyopadhyay and Dalui [16] investigated the effect of the menstrual cycle phases on endurance capacity and cardiorespiratory response. The participants in their study were 45 healthy sedentary eumenorrheic females aged 21–25 years. The participants were tested on three different occasions within a menstrual cycle, day 3 from the onset of menses (T1), day 10 from the onset of menses (T2), and between days 20 and 24 from the onset of menses (T3). They reported that VO_2max_, O_2_ pulse, maximum pulmonary ventilation, and endurance capacity were significantly lower on T2 than on other test occasions. Furthermore, while motivation is beyond the scope of this article, some studies reported similarity in post-test heart rate, the rate of perceived exertion, and the blood lactate values measured [31], indicating that motivation might have played a role in the results of the studies that found difference in performance between testing occasions [31].

The conflicting results observed in the literature have created a space of possibilities, calling for more research on the subject but also a new analysis technique in the field that could “reallocate of credibility across a space of candidate possibilities” [34]. While most of the studies used the approach of rejecting the null difference, we sought to use an approach that would allow not just to reject the difference of null but also to assess the credibility of the null value and to assess the acceptance of the null value [34]. Therefore, the purpose of this investigation was to examine the variation in strength and aerobic physical performances on different testing occasions in young eumenorrheic female college students within a menstrual cycle compared to the menstruation phase using the Bayesian hierarchical model.

## 2. Materials and Methods

### 2.1. Study Design

A repeated measure experimental design was developed and carried out to investigate the variation in physical performance from different testing occasions compared to the menstruation phase. This study was conducted between May and August 2018, where the participants were followed up for two menstrual cycles before the menstrual cycle they were tested in. The independent variables assessed in this study were the testing occasions, which, in this study, were four occasions from the onset of menses; on day 2 (T1), day 8 (T2), day 14 (T3), and day 21 (T4) (details provided under Section 2.3.1). Therefore, the dependent variables in the present investigation were the physical performance tests’ results.

### 2.2. Participants

To compare the results from the present investigation to other published investigations, and due to the difficulties of recruiting participants for menstrual studies, our goal of the minimum sample size was set based on published studies. Therefore, a minimum of 10 participants was the lowest number of participants that we could accept, which is in line with several other studies [10,14,17,23,24,27,28,31,35,36,37]. Hence, 12 eumenorrhea female college physical education students volunteered to participate in this study. The participants were 19.8 ± 0.8 (±SD) years old, with the body mass of 61.4 ± 11.6 kg, the height of 162.6 ± 5.1 cm, and BMI of 23.2 ± 3.8. All participants reported regular monthly menstrual cycles of 26–33 days during the two cycles before testing start. None reported having been taking oral contraceptives in their entire life (a female assistant researcher controlled the reporting of the onset of menses; see Section 2.3.1). To be included in this study, the menstrual cycles during the two cycles before testing took place had to be between 26 and 35 days [3]. None of the participants in this study reported any current or ongoing neuromuscular diseases or musculoskeletal injuries, and none was taking any dietary or performance-enhancing supplements that could have affected testing results during this study. None of the participants was an athlete, and their level of activity was limited to physical education classes and recreational activities. Informed written consent was obtained from all participants after a verbal and a written explanation of the experimental design and potential risks.

### 2.3. Procedure and Instruments

#### 2.3.1. Experimental Protocol

**Figure 1:**
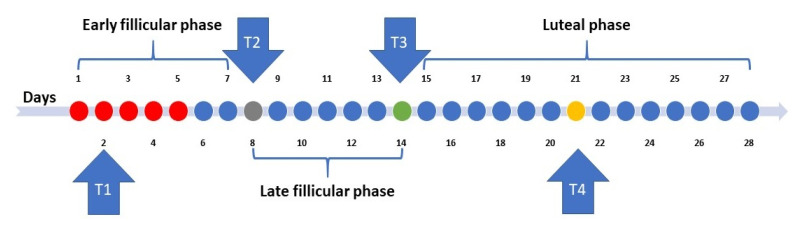
The classical model of the menstrual cycle and the timeline for data collection.

The main aim of the experimental protocol was to compare performance during the menstruation phase to other days within a menstrual cycle. The testing days in this study were spread over a month and the test was conducted within one menstrual cycle (i.e., between menstruation to menstruation phases). Therefore, and to compare to other studies and to provide adequate recovery between testing days [2,10,38], 4 test occasions were planned based on the classical model of the menstrual cycle (i.e., 28 days; Figure 1). Hence, the first performance test was carried out on the early follicular phase (i.e., menstruation phase) on day 2 (T1). Moreover, the second (T2; late follicular phase), the third (T3; ovulation phase), and the fourth (T4; mid-luteal phase) performance tests were conducted on days 8, 14, and 21 from the onset of menses [12,13,14,15,16,18,22,24]. To minimize experimental bias, due to participants’ familiarity with the tests, all tests were conducted randomly based on each participant’s onset of menses, which were coordinated with a female student assistant researcher. However, since the participants could not be blinded from their menstrual cycle, blinding of the researchers was, on the other hand, possible and necessary for the participants to feel comfortable participating. Therefore, the lead researchers (i.e., the authors) conducted the tests, and the coordinator (i.e., the female student assistant) coded the results according to the test occasion being tested. The labelling and results were handed to the lead researchers after all of the tests were completed.

#### 2.3.2. Anthropometry

The participants’ height and body mass were assessed on T1 when submitted to the testing laboratory. Height was first measured (barefoot) using a wall-mounted stadiometer (measuring between 6 and 230 cm with a graduation of 1 mm; Seca stadiometer model 222; Seca Medical Measuring Systems and Scales, Hamburg, Germany). The test person has to make three height reads to make sure that the stadiometer is probably mounted. Then body mass was assessed using the InBody body composition analyzer (model: InBody570; InBody Co., Ltd., Seoul, Korea). The validity and reliability of the InBody body composition analyzer were assessed earlier [39].

#### 2.3.3. Warm-Up and Testing Procedure

Before maximum strength testing, a general warm-up consisted of 5 min of low-intensity jogging to increase heart rate, blood flow, and deep muscle temperature. Then, there was a specific warm-up consisting of one set of 10 repetition maximum (RM) followed by one set of 5RM (the specific warm-up procedure was followed for both upper and lower body strength) [40]. The following test sequence was followed: (I) 1RM bench press using smith machine (model: SSM, Life Fitness, Rosemont, IL, USA); (II) 1RM leg press using plate-loaded linear leg press machine (model: SPLLLP, Life Fitness, Rosemont, IL, USA); (III) upper body muscle endurance using push-up to failure [41]; (IV) lower body muscle endurance using leg press with 60% of 1RM to failure [41]; (V) ccardiorespiratory fitness was assessed by running 1600 m (time trial performance) [42]. The effects of fatigue were prevented by providing the participants with >5 min of recovery between strength tests, and >1 h of recovery was provided between strength tests and the 1600 m time trial [40,41]. The participants were tested at the same time of day on each testing occasion (i.e., testing started at 10:00 a.m.) to avoid diurnal variations. However, in the incident where two or more participants had their onset of menses on the same day, the order of the tested participants was also the same on each testing occasion.

### 2.4. Statistical Analysis

**Figure 2:**
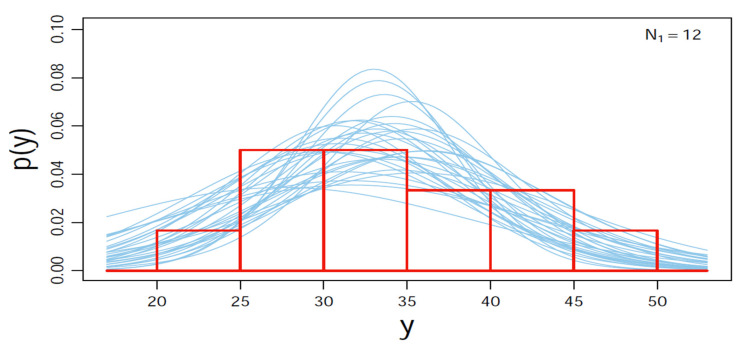
Example of the posterior predictive checks (PPC) of the 1RM bench press performance from the menstruation phase (T1) plotted and visually checked with the original data (the complete PPC plots from each analysis can be found in S1: results and diagnostics).

The choice of statistical analyses in this study was based on two main reasons. First, as indicated in the introduction, the different studies’ conflicting results create “suspicion and scepticism” [34]. Second, while most of the studies used the approach of rejecting the null difference, we sought to use an approach that would allow not just rejecting the null difference but also assessing the credibility of the null value and assessing the acceptance of the null value [34], i.e., the magnitude of the difference between central tendencies by estimating those magnitudes and assessing those estimates’ uncertainty [34]. While this data analysis approach is limited in sport science studies, it has been used in several physiological and psychological sport-related studies [43,44]. Therefore, all statistical analyses in this study were conducted using R package version 3.6.1 (R Core Team, Vienna, Austria) and RStudio version 1.3.1093 (RStudio Team, Boston, MA, USA). The Bayesian Estimation of differences in performance from the four testing occasions was assessed using the Bayesian hierarchical model (Bayesian Estimation Supersedes the *t*-Test (BEST)) with Markov Chain Monte Carlo (MCMC) simulation as a part of the model (BESTmcmc), which is integrated into JAGS package version 4.3.0 (Martyn Plummer, international agency for research on cancer, Lyon, France [45]). The R code (BEST.pdf (r-project.org, accessed on 20 May 2021)) provides the complete script, including the model specification and the graphics commands adapted from Kruschke [34]. The BESTmcmc simulation was carried out by running 1 “chain”, 1000 steps to “burn-in,” and 1st place as a number of “thin” with 100,000 credible parameter samples to save (sample size) for each variable in this study [34,46]. The outliers were fitted to the model by using the BEST *t* distribution of the data (i.e., fatter tails) [34]. The goal of the MCMC process is to generate an accurate and reliable representation of the posterior distribution; therefore, the values of the BESTmcmc simulation had to meet the criteria of representativeness and accuracy by examining the convergence of the MCMC algorithm, which was checked using the Brooks-Gelman-Rubin scale reduction factor (Rhat), which is 1 on convergence with values below 1.1, which are considered to be acceptable [47]. Furthermore, the effective sample size (n.eff) was checked by the results of n.eff; a value of n.eff > 10,000 is needed for stable estimates of 95% credible interval [46,48]. The posterior predictive distributions were plotted and visually checked with the original data to further check if the model has a reasonably good fit for the sample data (e.g., Figure 2 and Figure S1: results and diagnostics). Furthermore, since past studies vary in tests and measurements used to assess the effect of menstrual cycle on performance, establishing a prior distribution that meets the skeptical scientific audience from these studies would be difficult. Hence, to assure that the MCMC process of reallocation of credibility is close to the measured values and meets the skepticism of the scientific audience, the prior was set to = NULL. A NULL prior implicates that the prior is not too small to reflect the desired outcome; rather, it is broad, as described by Kruschke [34] (i.e., “standard deviation of the prior on µ were set to 1000 times the standard deviation of the pooled data and the mean M of the prior on µ is arbitrarily set to the mean of the pooled data”). The prior specification can be found in the model specification at BEST.pdf (r-project.org, accessed on 20 May 2021).

The descriptive statistics were reported as mean and standard deviations (SD) of the mean (Table 1). Furthermore, the differences in performance from the four test occasions were reported as Bayesian estimated difference of the mean of the posterior ± 95% high-density interval (HDI) and the estimated SD of the posterior ± 95% HDI of the estimated SD from all the participants on all variables in this study (Table 2). Furthermore, to be able to make an intuitive decision to accept or reject the null value, the decision-theoretic properties of the HDI + ROPE decision rule were adapted, considering the magnitude of the parameter value [49]. The decision rules followed in this study looked mainly at three possibilities; first, if the most credible parameter values (95% HDI) are sufficiently away from the null value (null value can be rejected); the second, if the most credible parameter values are close to the null value (null value can be accepted); and third, if neither the first nor the second is met, the decision rule would be undecided, which indicates that more research is still required (more information regarding the decision rules can be found in Kruschke [49]). In this study, the ROPE was defined as half the smallest effect size (i.e., *d* ± 1) [34,46,49,50].

## 3. Results

**Figure 3:**
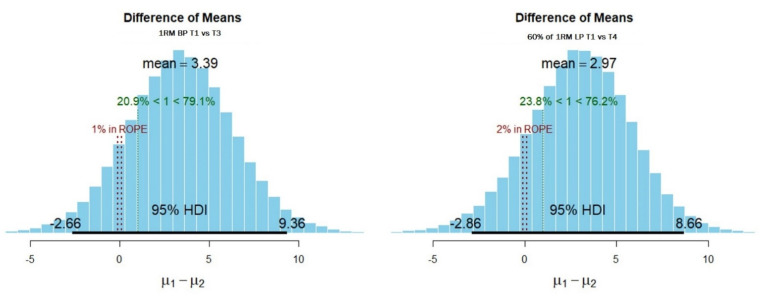
The most credible parameter values (95% HDI) for 1RM bench press (BP) from T1 vs. T3 and leg press (LP) with 60% of 1RM to failure from T1 vs. T4.

The Bayesian estimated difference from the four testing occasions neither showed that the most credible parameter values (95% HDI) were sufficiently away from the null value nor showed that the most credible parameter values are close to the null value (Table 2). Nevertheless, 1RM bench press from T1, compared to T3, and the number of repetitions to exhaustion from leg press using 60% of 1RM from T1, compared to T4, showed a notable small difference indicated by effect size > 0.4 (Figure 3; Table 2).

## 4. Discussion

This investigation’s main findings revealed no apparent performance differences between the menstruation phase and the testing results from the other three testing occasions (Table 2). The reduction in estrogen has been shown to decrease muscle performance-related organs such as mitochondrial function, membrane microviscosity, and others [7,8]. Since estrogen is lowest during the menstruation phase [8], it was expected that T1 would decrease performance compared with T2, T3, and T4 [1]. However, in contrast to our expectations, the results of this investigation revealed no indications of notable harm or benefits to performance between the menstruation phase and T2, T3, and T4 when examined using eumenorrhea female college students with a menstrual cycle that lasts around 26–35 days. This could be further explained by the fact that the stored muscle glycogen has not been altered by the hormonal fluctuations across the menstrual cycle, indicating that the energy supplies between the four test occasions were also unaltered [23].

### 4.1. Aerobic Capacity Time Trial Performance

The newly published studies confirm the findings of this study, e.g., Gordon et al. [23], who investigated the effects of the menstrual cycle phase in a group of regularly menstruating participants. The participants were similar to those in this study. The authors concluded that menstrual cycle phases in the regularly menstruating population did not affect VO_2max_ or parameters used in its classification (i.e., cardiac output, stroke volume, and heart rate). Furthermore, their findings were supported by the fact that the manifestation of a plateau achieved at the VO_2max_ is a function of the availability and utilization of the high-energy phosphates (ATP-PC) stored in the working muscles [23,51]. Therefore, the non-statistical difference in cardiac output and the demand for oxygen at a cellular level [23], combined with no changes in peak anaerobic power between test occasions within a menstrual cycle [18], could explain the results of no differences. Studies similar to that conducted by Gordon et al. [23] have been reported in female soccer players [25], and physically active eumenorrheic females [14], showing that endurance performance was similar across the testing occasions within a menstrual cycle and was explained by the no changes observed in power output, heart rate, peak Vo_2_, and maximal blood lactate.

Similarly, Vaiksaar et al. [15] confirmed no differences in power output, heart rate, oxygen consumption, carbon dioxide production, minute ventilation, mean respiratory exchange ratio, and ventilatory equivalents of O_2_ between the testing occasions within a menstrual cycle. Contrary to these findings, Bandyopadhyay and Dalui [16] reported statistically significantly higher measures of VO_2max_, O_2_ pulse, maximum pulmonary ventilation, and endurance capacity in test days 10 and 20–24 compared with menstruation phase (i.e., test day 3), and higher resting heart rate from test days 20–24 compared with test day 10 and menstruation phase. The findings were attributed to the autonomic nervous system activity, namely, the increase in the sympathetic nervous system activity in the luteal phase in response to hormonal change. This increase indicates an increase in respiratory muscles’ needs of O_2_ uptake. However, this increase in demand could have caused the noticed increase in the measured variables in test day 20–24 (i.e., luteal phase) [16]. These results are in conflict with the results from Gordon et al. [18,23], where it was reported that the VO_2max_ is a function of the availability and utilization of the ATP-PC stored in the muscles combined with no changes observed in maximal cardiac output and no increase in demand of oxygen at a cellular level within the engaged muscle.

In contrast to Gordon et al. [23] and Bandyopadhyay and Dalui [16], Julian et al. [31] reported a trend toward statistically significant difference in Yo-Yo Intermittent endurance test where the distance covered during test days 21–22 was notably less than in test days 5–7 from the onset of menses. They attributed the reduction in performance in test days 21–22 (i.e., luteal phase) to an increase in body temperature, which is thought to limit players’ capability to perform a prolonged exercise and further cause an increase in stress on the cardiovascular system. While Julian et al. [31] attribute the decline in performance to increased stress on the cardiovascular system, Bandyopadhyay and Dalui [16] attribute the opposite due to the same factor causing an increase in sympathetic nervous system activity. It is worth noting that Julian et al. [31] mention that motivation might have played a role in the results indicated by the similarity in posttest heart rate, the rate of perceived exertion, and the blood lactate values measured. Furthermore, it is worth noting that the pre-exercise heart rate in Julian et al. [31] was higher in test days 21–22 compared with test days 5–7, which is in line with the results from the Bandyopadhyay and Dalui [16] study. Interestingly, Tounis et al. [25] attributed the lack of differences between test days to the fact that the participants did not report premenstrual and menstrual symptoms. Hence, those arguments could direct future studies to examine the interaction effect between menstruation cycle, motivation, and autonomic nervous system activity on physical performance.

However, in this study, we measured time trial performance by asking the participants to run 1600 m in the shortest time possible. This approach in testing aerobic performance is well documented for its validity and reliability in measuring cardiorespiratory fitness [42]. Other studies using time trials to assess performance differences on different occasions within a menstrual cycle also concluded various results. For example, Forsyth and Reilly [36] investigated the effect of the menstrual cycle phase on 2000 m rowing ergometry performance on 10 eumenorrheic females. They reported a higher exercise intensity, heart rate, and oxygen consumption at 4 mmol·L^−1^ blood lactate concentration from test days 18–23 compared with test days 6–10 from the onset of menses. However, there was no difference in time trial performance between the two occasions. While the difference was small in the 2000 m rowing ergometry test (i.e., 3 s), that 3 s in the difference can be seen as large in a highly trained athlete [52].

Nevertheless, the participants in the study by Forsyth and Reilly [36] were recreational females. However, the higher performance indicator values obtained at 4 mmol·L^−1^ blood lactate concentration seems to have a trivial influence on the performance results. Hence, in line with Gordon et al. [23], this could be due to the fact that exercises relying mainly on the ATP-PC might not be affected by hormonal fluctuations during the menstrual cycle. Forsyth and Reilly [36] explained the small difference by the large variability in the participants’ results from the 2000 m time trial. Similar results with explanations similar to those by Gordon et al. [23] were also reported using a 16 km time trial on a eumenorrheic female after carbohydrate loading [53].

On the other hand, the estrogen levels are higher between days 18 and 23 (i.e., mid-luteal phase) and have a tendency to decrease the utilization of glycogen and reduce blood lactate concentration [7]. Campbell et al. [35] tested the hypothesis of glucose ingestion during exercise, which might eliminate this limiting factor. Even though the authors reported that glucose rates of appearance and disappearance may be higher during the first few days from the onset of menses (i.e., follicular phase) compared with days 18–29 of the menstrual cycle (i.e., mid-luteal phase), there were no time trial performance differences between the testing occasions. Furthermore, Oosthuyse et al. [37] conducted a time trial on two groups of eumenorrheic females, where one is trained and the second is untrained. They measured the time used to cover 15 and 30 km on both groups on 3 test occasions: namely, the first test, in days 2–7 (i.e., early follicular), the second test in days 10–14 (i.e., late follicular), and days 14–24 (i.e., mid-luteal phases) from the onset of menses. They reported no statistically significant difference in the finishing time between the three test occasions on both groups. Combining the two groups revealed, however, a trend toward better performance in the second test occasion (i.e., test days 10–14) compared with the menstruation phase (i.e., test days 2–7). The high estrogen concentrations explained improved performance during the second test occasion compared with the first test. However, compared with the study by Forsyth and Reilly [36], and considering the time trial variation between the two studies, one could expect that the difference observed in Oosthuyse et al. [37] would indicate a large effect taking into account the higher exercise intensity, higher heart rate, and higher oxygen consumption at 4 mmol L^−1^ blood lactate concentration in test days between 18 and 23. All these studies combined could explain the results of this study where there were no clear indications as to whether the menstruation phase would affect performance compared to the other testing days within a menstrual cycle; this was indicated by the Bayesian estimated difference from the 4 testing occasions, which neither showed that the most credible parameter values (95% HDI) are sufficiently away from the null value nor showed that the most credible parameter values are close to the null value (Table 2), indicating that no decision can be made yet regarding the effect of the menstrual cycle phase on the performance tests used in this study.

### 4.2. Maximal Strength or Strength Endurance

The results of the present study did not show any meaningful differences in maximal strength or strength endurance tests for both the lower and the upper body. However, the results showed a small effect on upper body maximal strength (Figure 3; Table 2) measured as 1RM in the bench press (T2 vs. T3) and small effect size on lower body strength endurance (Figure 3; Table 2) using leg press (T2 vs. T4). The findings in this study are consistent with the finding of a newly published study by Romero-Moraleda et al. [28], who investigated the effect of the menstrual cycle on strength and power performance in 13 eumenorrheic females. The results of their investigation revealed that the 1RM of lower body (i.e., squat exercise) muscle force (N), velocity (m/s), and power output (W) values was almost unchanged between the three testing occasions. However, the authors reported that at 60% of 1RM, the test conducted around day three after the onset of menses had the highest values among the three test occasions (namely, test days ~13 and ~21). In our study, the highest values were observed in T2 (i.e., test on day 8). While our findings regarding the timing of the highest value are not in line with Romero-Moraleda et al. [28], the findings from this investigation support the hypothesis presented by Smith et al. [54], which suggests that higher values in strength performance can be observed because estradiol (which is one of three estrogen hormones) is highest at the late follicular phase (i.e., test days between 8 and 14) and is documented to be associated with enhanced cortical excitation compared with other timings during the menstrual cycle.

Furthermore, this theory was further supported by a later study by Rodrigues et al. [27]. They reported a statistically significantly greater maximal voluntary contraction 2–3 days after the menstruation has stopped, compared with the menstruation and pre-menstruation phases, which is further in line with the results reported by Tenan et al. [20]. However, in line with the results of this investigation, most studies did not report statistically significant differences between test occasions using anaerobic power tests [12,13,14,21,24,25,26,31,33].

Although the result from this investigation is in line with several of the reported investigations, this study is not without limitations. Due to the conservative culture where this research was conducted, a major limitation of the present study was its inability to conduct blood sampling, which might have aided in discussing the results against a wider range of published research. Another limitation in line with other published studies was the sample size; this study’s initial sample was 12 eumenorrheic physical education students. However, this limitation was compensated by running the BESTmcmc simulation using 1 “chain”, 1000 steps to “burn-in”, and 1st place as a number of “thin” with 100,000 samples to save and a prior = NULL for each variable measured in this study. However, one of the limitations regarding Bayesian estimation is the lack of similar studies using the same statistical approach, which makes it difficult to compare. Hence, we chose to attach the data source as a supplementary where other researchers can examine and compare based on the statistical methods of their choice.

## 5. Conclusions

It can be noted from this study and other published studies that the effect of the menstrual cycle varies from one study to another. However, this study, to the best of the authors’ knowledge, is the first study looking at the difference in physical performance tests across menstrual cycles using the Bayesian estimated difference on a simulated large sample with the decision-theoretic properties of the HDI + ROPE decision rule. Therefore, and based on the HDI + ROPE decision rule, the results of this study could not decide whether or not there are any effects of the menstruation phase (i.e., T1) on physical performances compared to other test occasions (i.e., T2, T3, and T4) within a menstrual cycle. Due to the sensitivity of such studies, it is difficult to recruit a large sample. Therefore, we believe that more research is needed with authors making their source data available for other researchers to conduct a proper meta-analysis on a large data for better addressing this issue. Nevertheless, based on the results from this study and published studies combined with the explanations provided, the authors would recommend that future research investigate and profile motivation and autonomic nervous system activity during the bleeding phase and examine the interaction effect of the three on performance compared to the other testing occasions.

## Figures and Tables

**Table 1:** Descriptive statistics of the results from the four testing occasions (mean ± standard deviations (SD)).

Variable	T1	T2	T3	T4
Body mass (kg)	61.1 ± 11.7	61.2 ± 11.8	61.0 ± 11.6	61.4 ± 11.6
1RM bench press (kg)	33.2 ± 7.4	35.4 ± 7.1	32.1 ± 5.6	33.7 ± 8.7
1RM leg press (kg)	98.1 ± 30.2	104.7 ± 36.0	95.3 ± 3.98	101.6 ± 35.7
Push-ups (*n*)	20.3 ± 7.1	21.3 ± 9.7	21.2 ± 7.2	19.3 ± 7.6
Leg press (*n*)	15.3 ± 6.4	15.9 ± 4.8	16.0 ± 8.4	13.4 ± 7.7
1600 m (s)	613 ± 104	589 ± 80	617 ± 94	612 ± 76

T1 = day 2 from the onset of menses; T2 = day 8 from the onset of menses; T3 = day 14 from the onset of menses; T4 = day 21 from the onset of menses; 1RM = one repetition maximum (source data can be found in S1: source data).

**Table 2:** Bayesian estimated performance differences between the four testing occasions (the complete results with figures can be found in S1: results and diagnostics).

Variable	Mean Deff. ± (95% HDI)	SD Diff. ± (95% HDI)	ES	Rope (OR%)
Body mass (T1 vs. T2) (kg)	−0.14 (−11.2–10.7)	0.22 (−9.9–9.7)	−0.01	51.2 < 0 < 48.8
Body mass (T1 vs. T3) (kg)	0.02 (−10.7–10.9)	−0.056 (−9.5–9.6)	0.02	49.9 < 0 < 50.1
Body mass (T1 vs. T4) (kg)	−0.31 (−11–10.7)	0.05 (−9.4–9.9)	0.00	52.3 < 0 < 47.7
Body mass (T2 vs. T3) (kg)	0.12 (−10.5–11.4)	−0.09 (−9.6–9.8)	0.02	49.2 < 0 < 50.8
Body mass (T2 vs. T4) (kg)	−0.14 (−11.1–10.9)	0.05 (−9.3–10.1)	−0.01	51.4 < 0 < 48.6
Body mass (T3 vs. T4) (kg)	0.29 (−11–10.7)	0.04 (−9.9–9.3)	0.02	52.1 < 0 < 47.9
1RM BP (T1 vs. T2) (kg)	−2.13 (−9.1–4.7)	0.58 (−5.6–6.3)	−0.27	73.7 < 0 < 26.3
1RM BP (T1 vs. T3) (kg)	1.31 (−4.9–7.5)	1.52 (−3.2–7.53)	0.17	33 < 0 < 67
1RM BP (T1 vs. T4) (kg)	−0.16 (−7.8–7.4)	−1.2 (−8.1–5.3)	−0.03	51.5 < 0 < 48.5
1RM BP (T2 vs. T3) (kg)	3.39 (−2.7–9.4)	1.28 (−3.4–7.1)	0.44	12.7 < 0 < 87.3
1RM BP (T2 vs. T4) (kg)	1.95 (−5.5–9.4)	−1.41 (−8.0–4.9)	0.22	29.2 < 0 < 70.8
1RM BP (T3 vs. T4) (kg)	−1.42 (−8.2–5.4)	−2.64 (−9.4–2.5)	−0.16	66.4 < 0 < 33.6
1RM LP (T1 vs. T2) (kg)	−5.28 (−36.6–24.9)	−3.66 (−31.5–23.4)	−0.17	64 < 0 < 36
1RM LP (T1vs. T3) (kg)	3.79 (−28.9–37.4)	−7.59 (−39.7–18.1)	0.15	40.7 < 0 < 59.3
1RM LP (T1 vs. T4) (kg)	−2.87 (−34.3–28)	−4.68 (−33.5–20.6)	−0.03	57.4 < 0 < 42.6
1RM LP (T2 vs. T3) (kg)	9.3 (−25.9–43.5)	−3.97 (−36–25.8)	0.26	28.8 < 0 < 71.2
1RM LP (T2 vs. T4) (kg)	2.59 (−30.4–35.4)	−0.76 (−31.1–27.8)	0.11	43.4 < 0 < 56.6
1RM LP (T3 vs. T4) (kg)	−6.54 (−41.5–29.6)	1.9 (−26.8–35.5)	−0.16	64.7 < 0 < 35.3
PU (T1 vs. T2) (*n*)	−0.66 (−8.5–7.2)	−2.31 (−10–3.8)	−0.10	56.7 < 0 < 43.3
PU (T1 vs. T3) (*n*)	−0.73 (−7.1–5.7)	−0.34 (−5.9–5.7)	−0.09	59.1 < 0 < 40.9
PU (T1 vs. T4) (*n*)	0.62 (−6.1–7.5)	−0.70 (−6.8–5.2)	−0.09	42.9 < 0 < 57.1
PU (T2 vs. T3) (*n*)	−0.11 (−8.0–7.6)	2.38 (−4.0–9.8)	−0.03	51.5 < 0 < 48.5
PU (T2 vs. T4) (*n*)	1.31 (−6.9–9.3)	1.84 (−4.8–9.4)	0.13	37.5 < 0 < 62.5
PU (T3 vs. T4) (*n*)	1.34 (−5.4–8.3)	−0.63 (−6.8–5.3)	0.19	34.8 < 0 < 65.2
LP (T1 vs. T2) (*n*)	−0.92 (−5.8–3.4)	0.74 (−3.6–5.7)	−0.17	65.9 < 0 < 34.1
LP (T1 vs. T3) (*n*)	−0.46 (−7.2–6.2)	−2.22 (−9.0–3.3)	−0.06	54.6 < 0 < 45.4
LP (T1 vs. T4) (*n*)	2.26 (−3.4–8.0)	−1.16 (−7.0–4.0)	0.34	20.7 < 0 < 79.3
LP (T2 vs. T3) (*n*)	0.21 (−6.4–6.5)	−3.26 (−9.8–1.3)	0.03	46.6 < 0 < 53.4
LP (T2 vs. T4) (*n*)	2.97 (−2.9–8.7)	−2.27 (−8.0–2.5)	0.41	14.7 < 0 < 85.3
LP (T3 vs. T4) (*n*)	2.61 (−4.6–10.1)	1.03 (−5.5–7.8)	0.29	22.9 < 0 < 77.1
1600 m (T1 vs. T2) (s)	24.9 (−65.5–112)	21.1 (−48.6–103)	0.26	28.4 < 0 < 71.6
1600 m (T1 vs. T3) (s)	−2.60 (−95.6–93.8)	8.33 (−69.8–94)	−0.02	52.2 < 0 < 47.8
1600 m (T1 vs. T4) (s)	0.04 (−88.8–85.4)	29.4 (−38.9–111)	0.04	49.7 < 0 < 50.3
1600 m (T2 vs. T3) (s)	−27.5 (−113–53.6)	−12.1 (−87.8–55.5)	−0.32	75.2 < 0 < 24.8
1600 m (T2 vs. T4) (s)	−25.1 (−99.1–50.8)	5.38 (−58.7–69.8)	0.30	75.4 < 0 < 24.6
1600 m (T3 vs. T4) (s)	2.45 (−76.6–85.5)	17.8 (−49.2–91.9)	0.04	47.6 < 0 < 52.4

BP = Bench press; LP = leg press; PU = Push-ups.

## Data Availability

The data supporting the reported results are available online as Supplementary Materials at https://www.mdpi.com/article/10.3390/sports9090130/s1.

## Supplementary Materials

The following are available online at https://www.mdpi.com/article/10.3390/sports9090130/s1, S1: results and diagnostics; S1: source data; S1: results using frequentist methods.
