# Supplementary material for: Bayesian Estimation of the Variation in Strength and Aerobic Physical Performances in Young Eumenorrheic Female College Students during a Menstrual Cycle
Source: Sports (Basel). 2021 Sep 17;9(9):130. doi: 10.3390/sports9090130 (PMC8472434; doi:10.3390/sports9090130)

Group 1 = Test day 2

Group 2: Test day 8

S1: Results and diagnostics

Body mass comparison

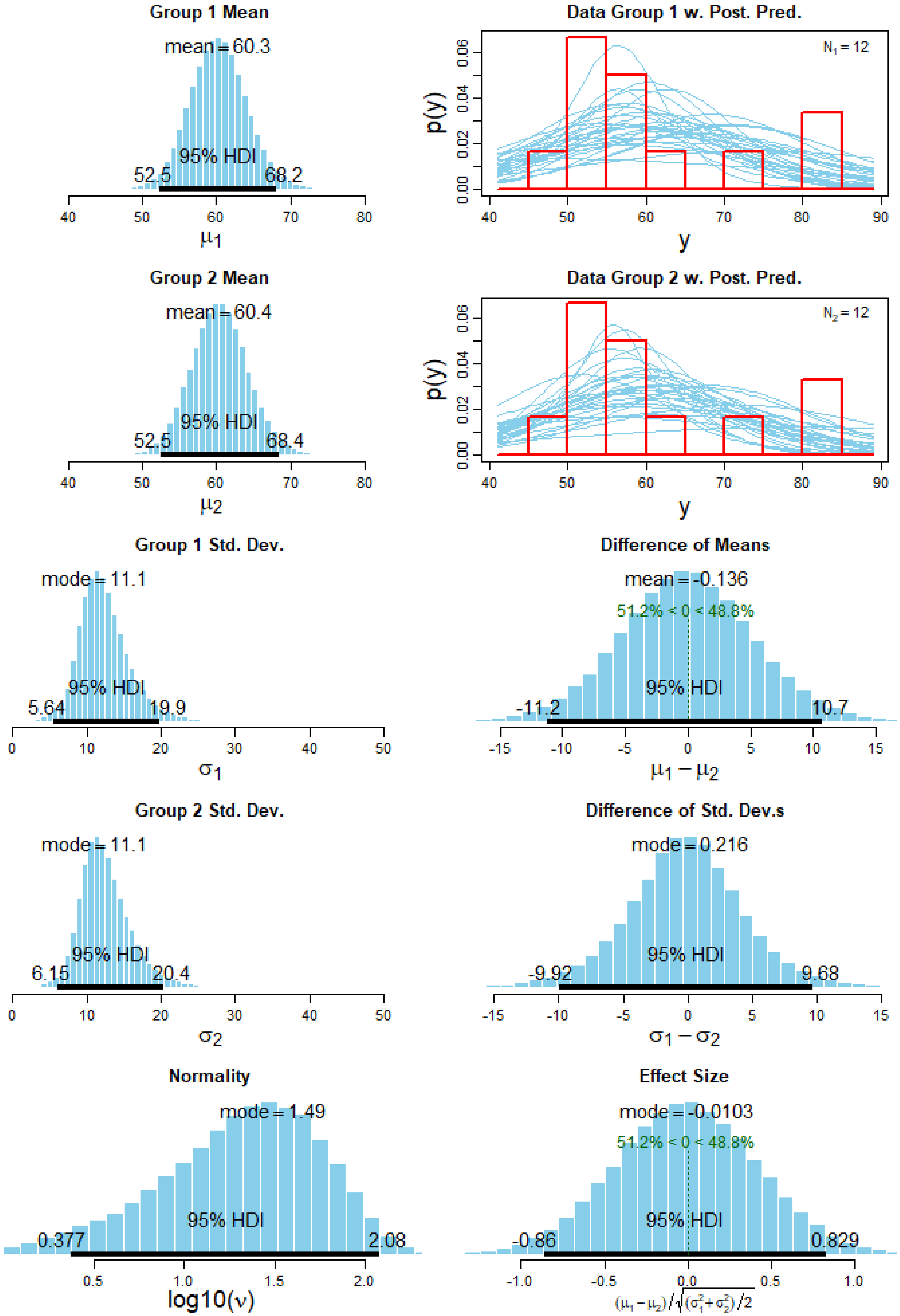

Group 1 = Test day 2

Group 2: Test day 21

Body mass comparison

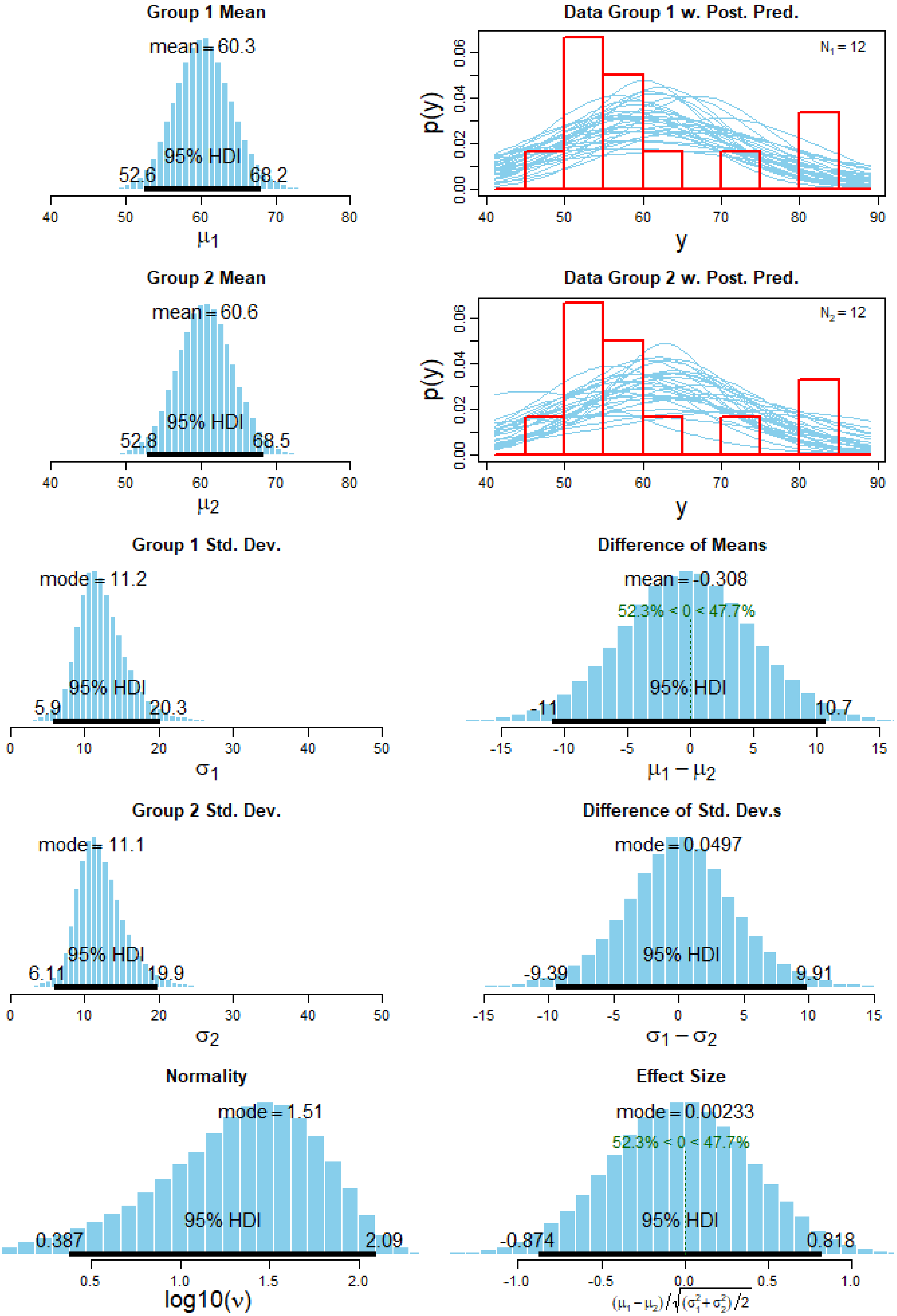

Group 1 = Test day 2

Group 2: Test day 14

Body mass comparison

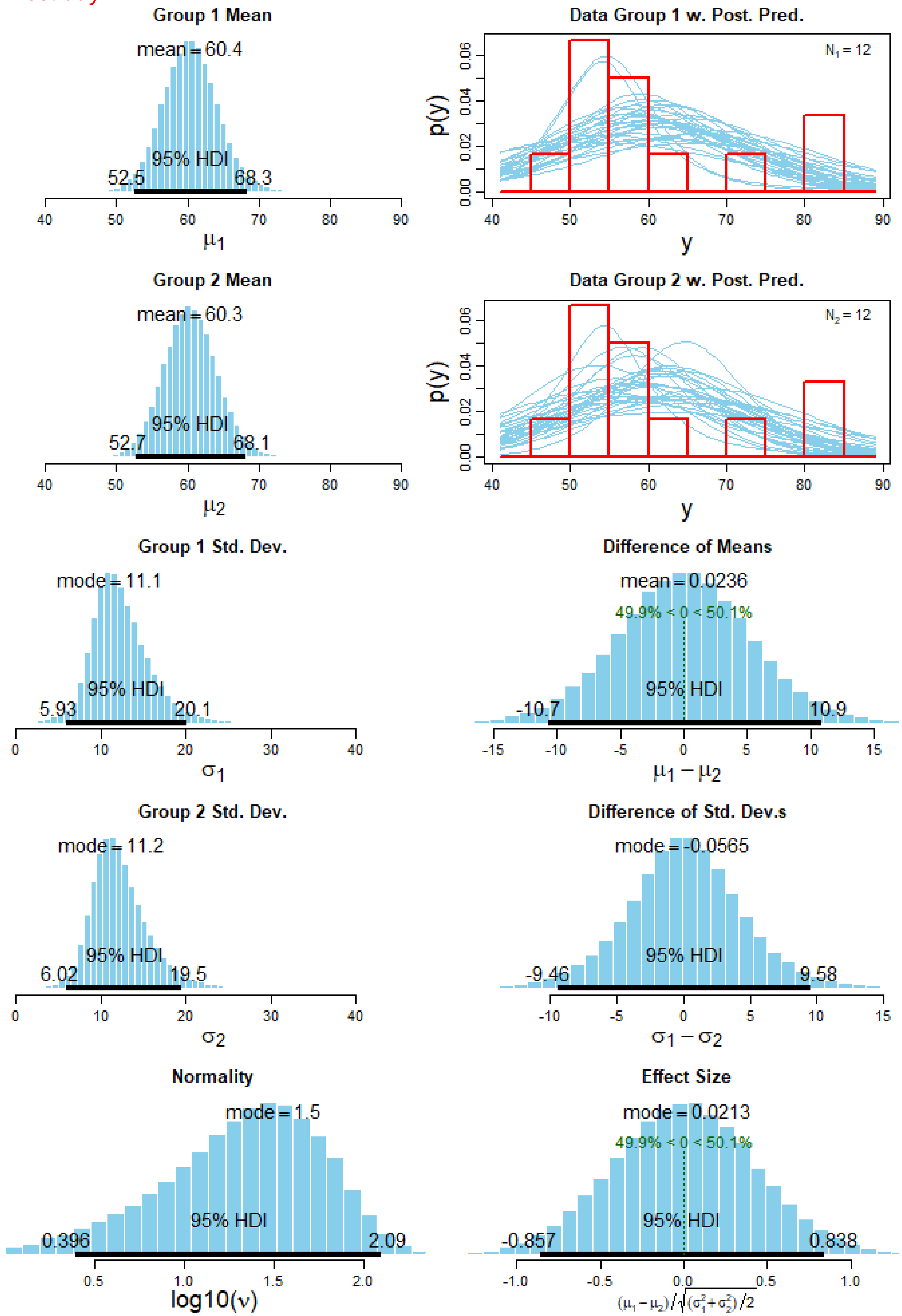

Group 1 = Test day 8

Group 2: Test day 21

Body mass comparison

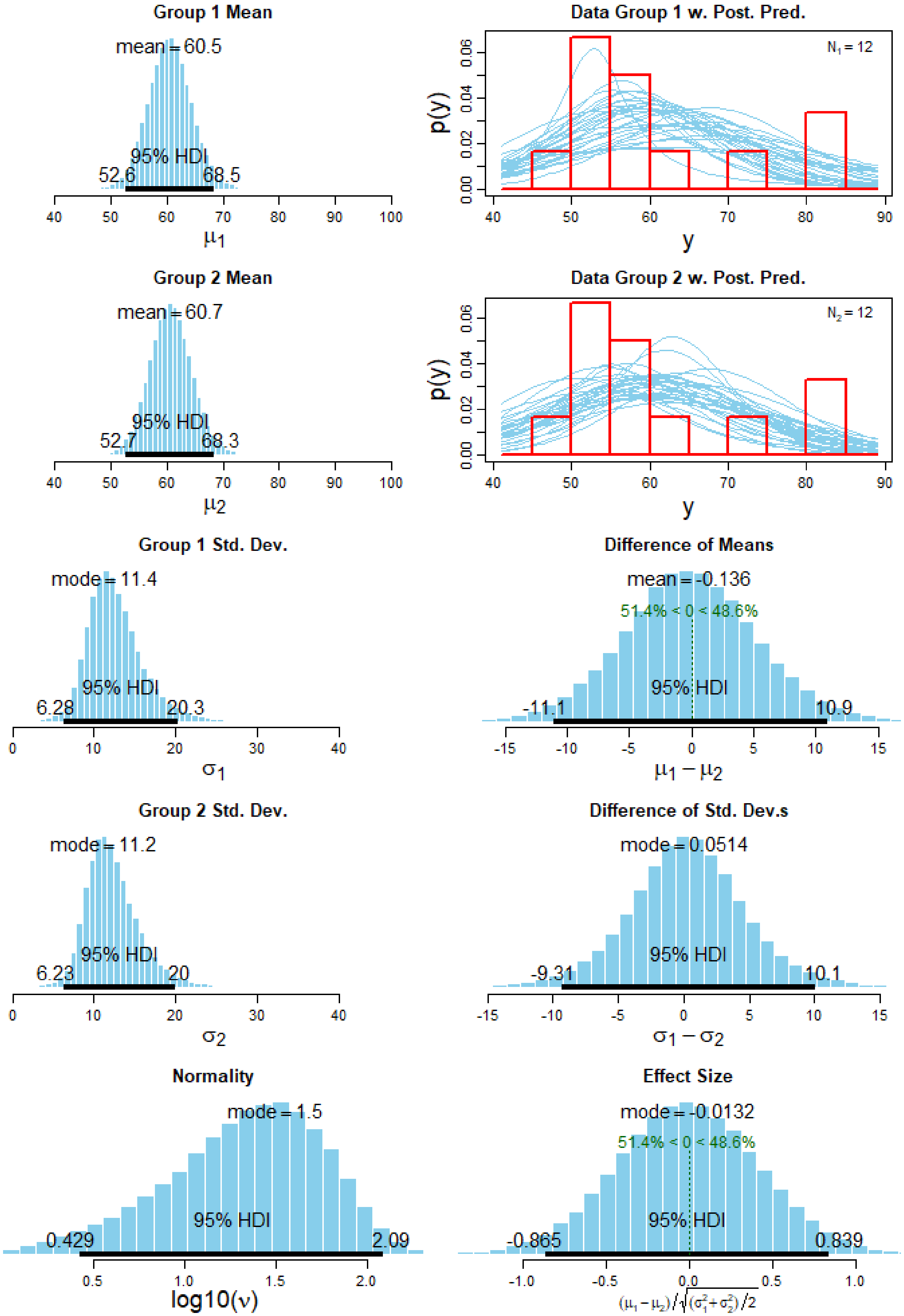

Group 1 = Test day 8

Group 2: Test day 14

Body mass comparison

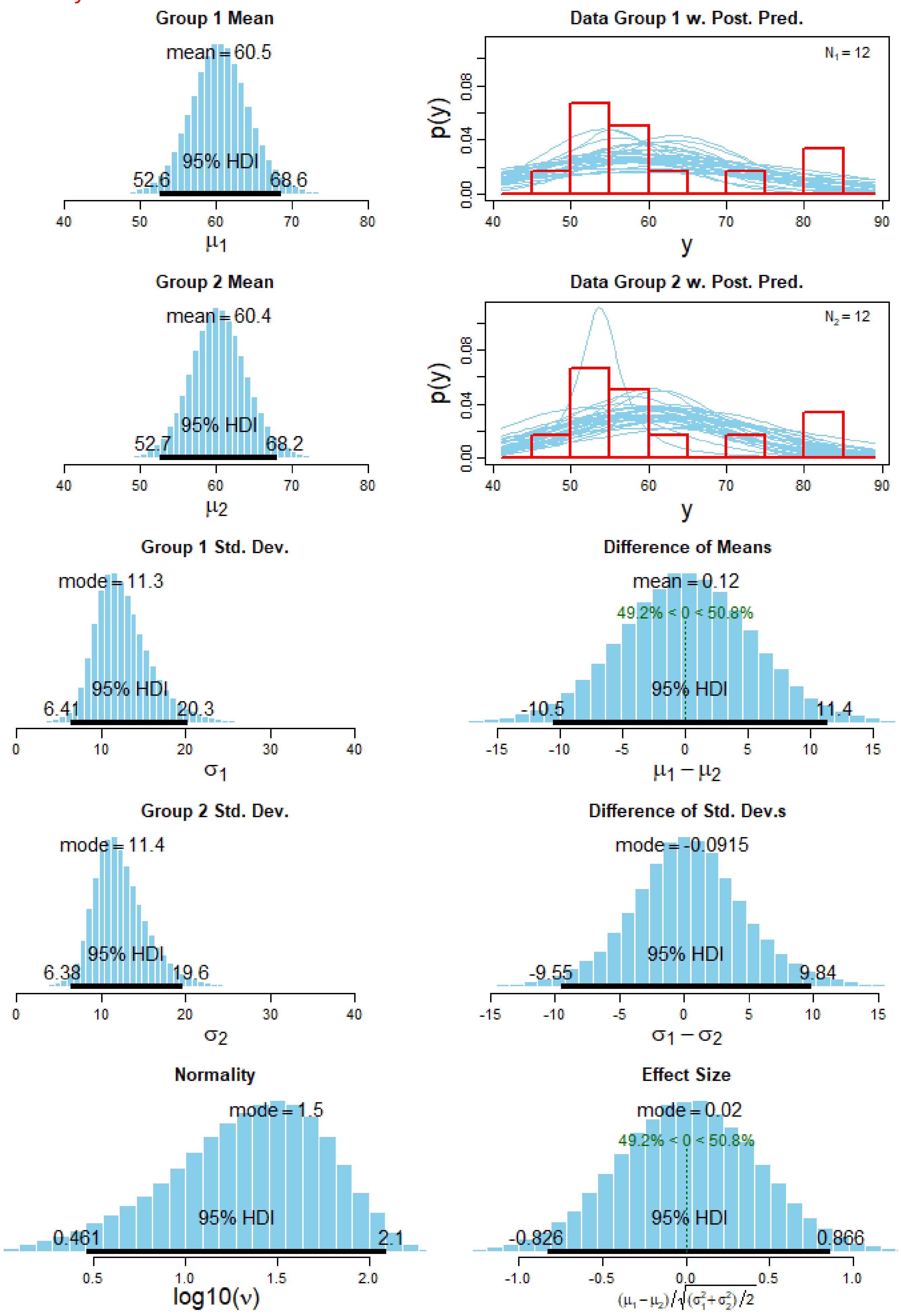

Group 1 = Test day 14

Group 2: Test day 21

Body mass comparison

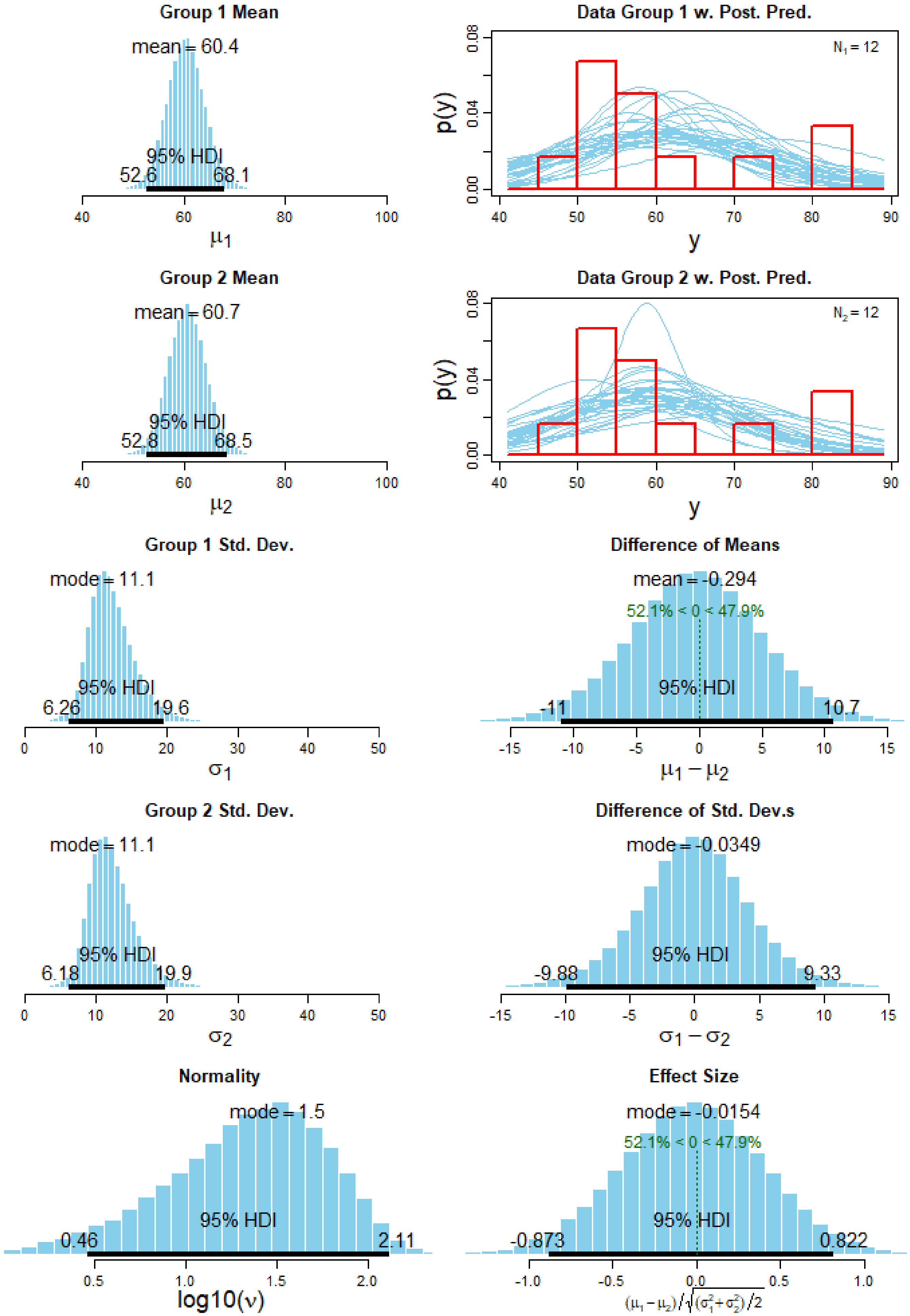

Group 1 = Test day 8

Group 2: Test day 21

1RM Bench Press

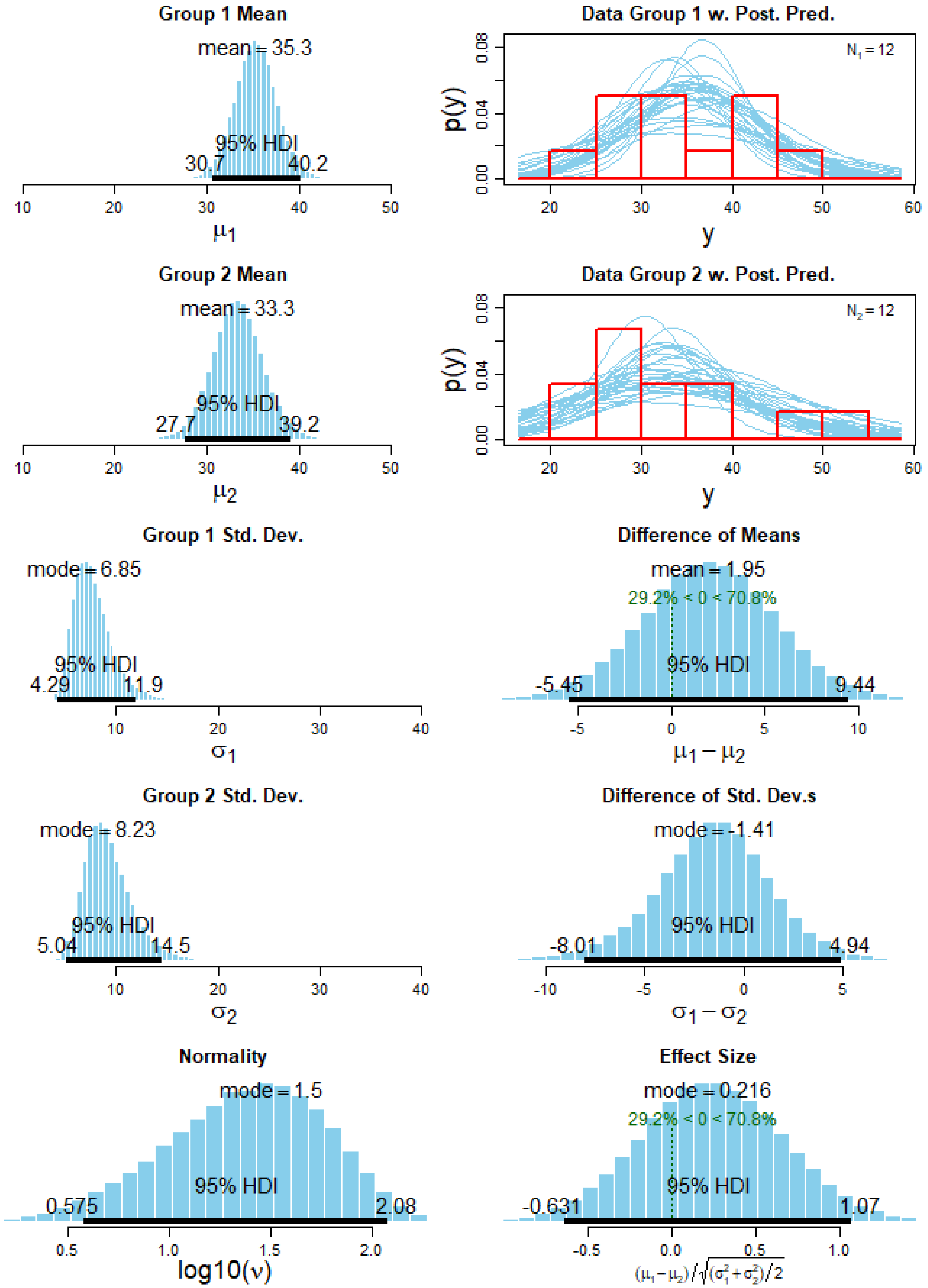

Group 1 = Test day 8

Group 2: Test day 14

1RM Bench Press

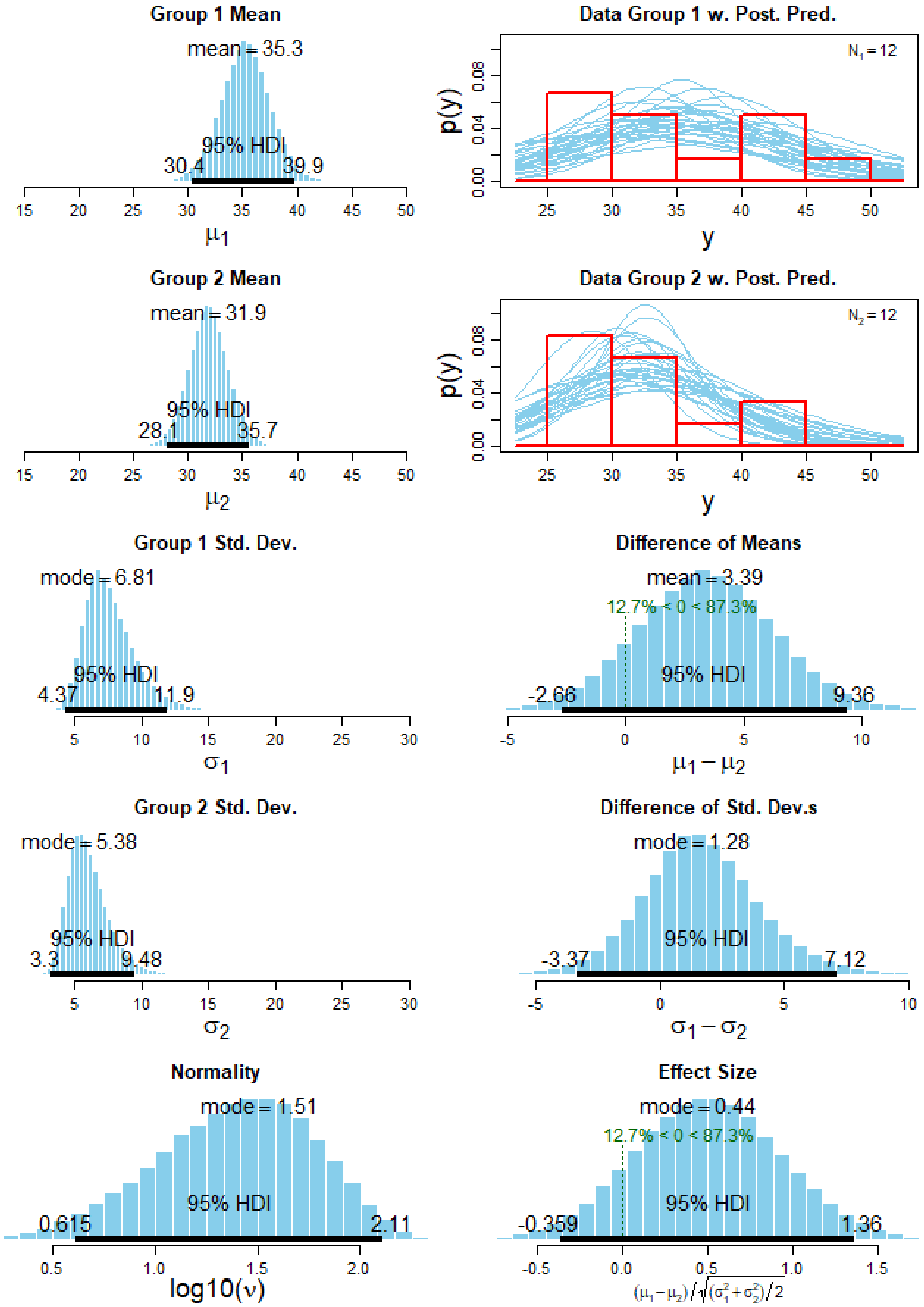

Group 1 = Test day 2

Group 2: Test day 8

1RM Bench Press

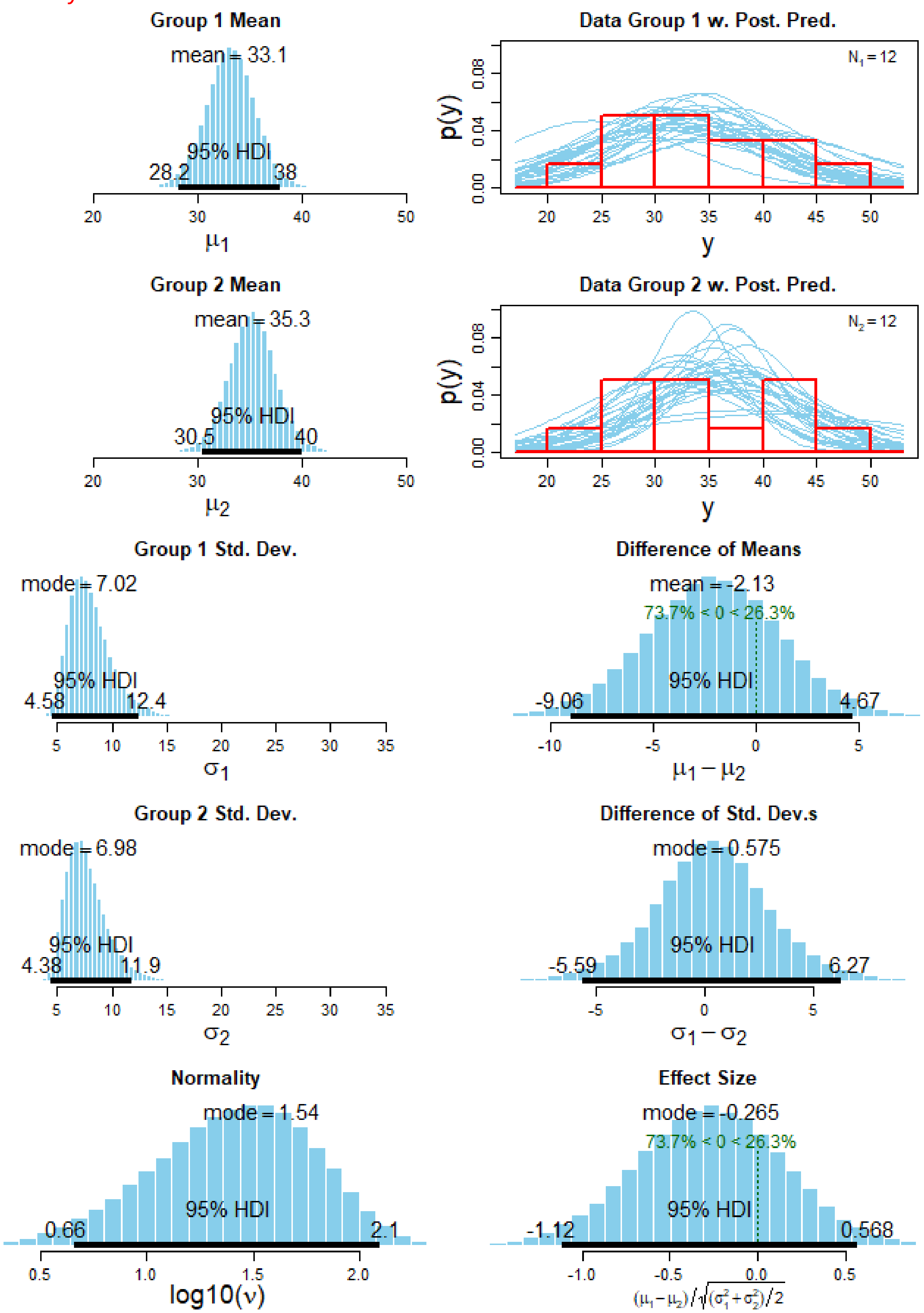

Group 1 = Test day 2

Group 2: Test day 21

1RM Bench Press

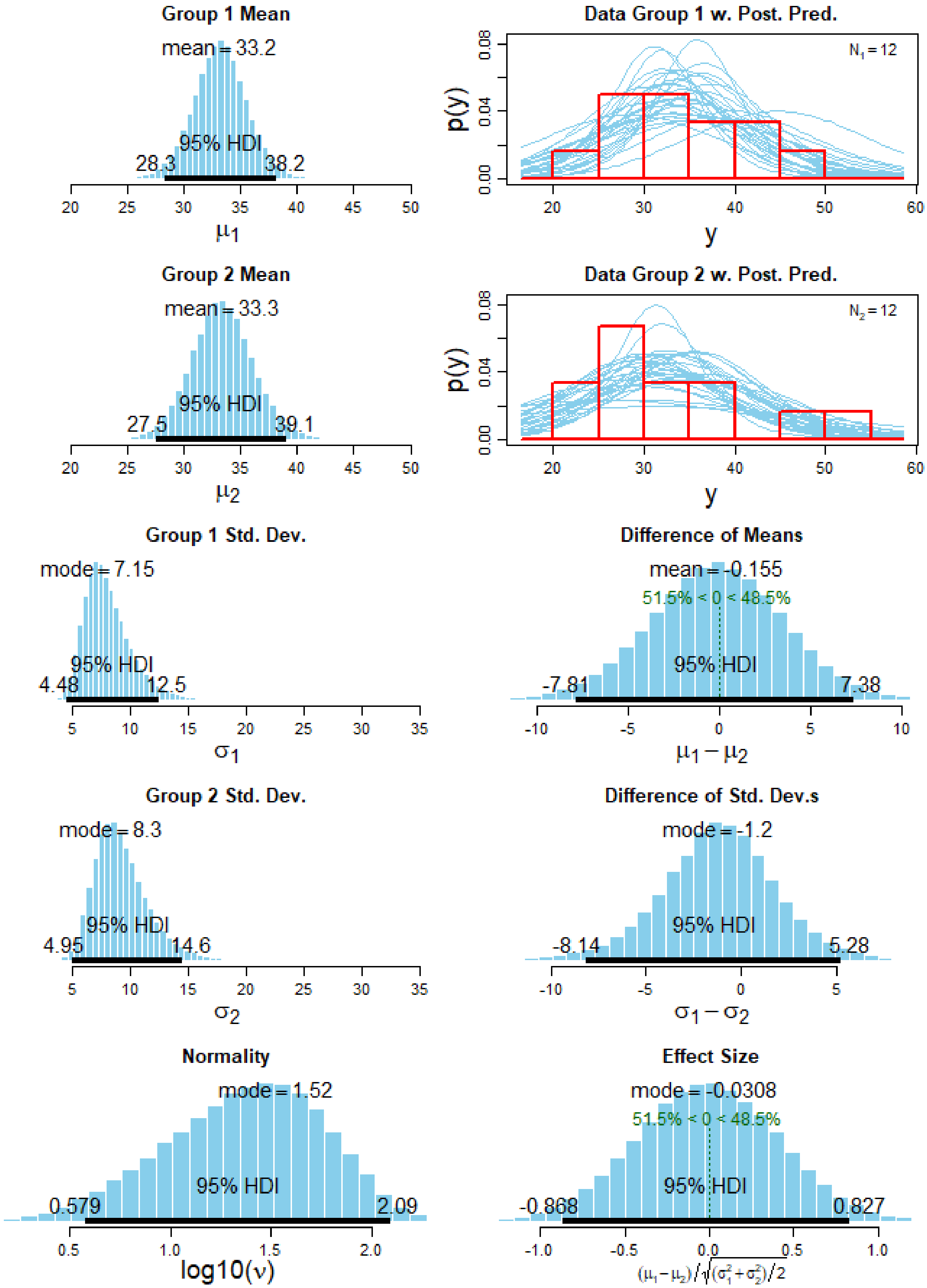

Group 1 = Test day 2

Group 2: Test day 14

1RM Bench Press

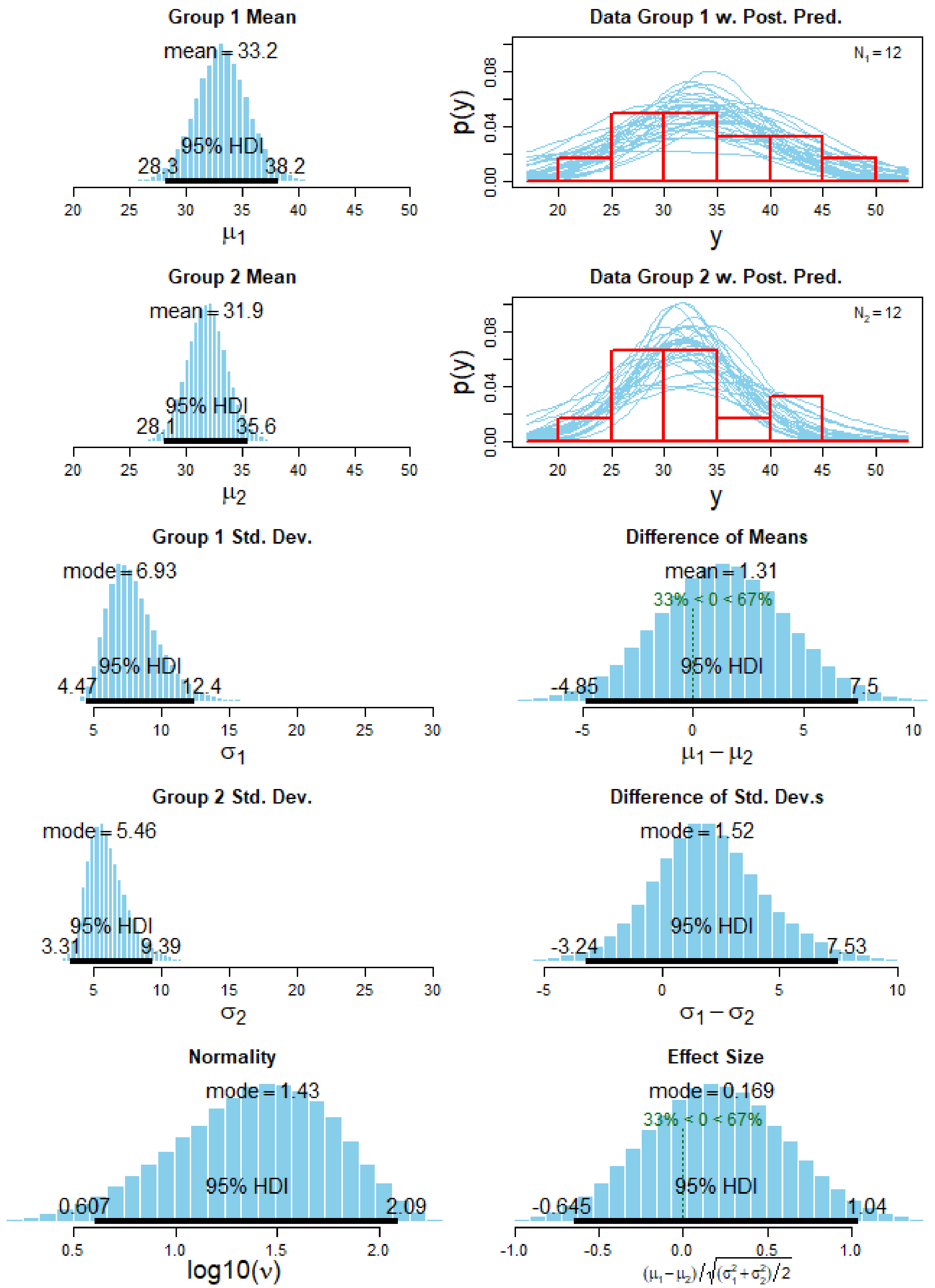

Group 1 = Test day 14

Group 2: Test day 21

1RM Bench Press

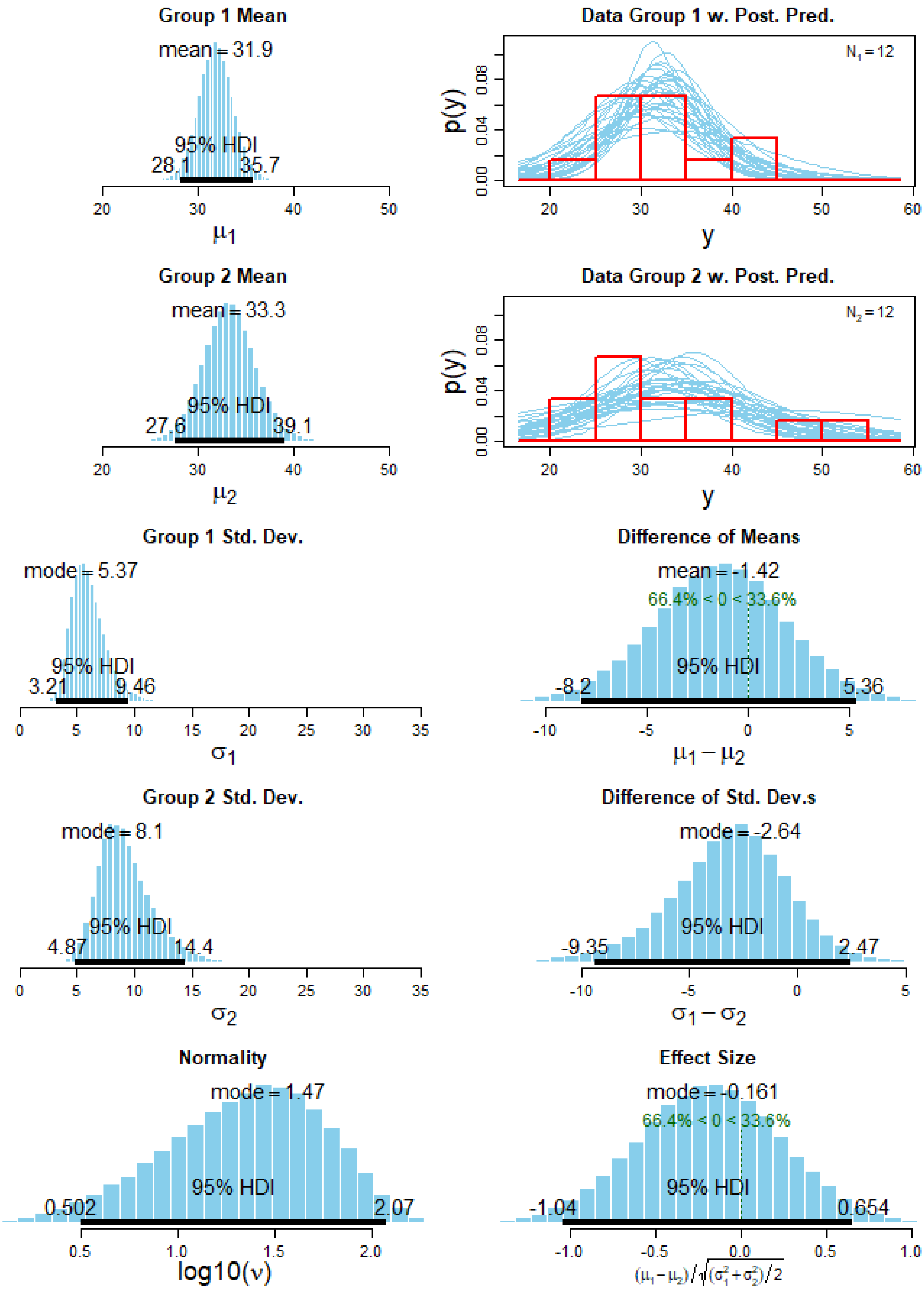

Group 1 = Test day 8

Group 2: Test day 21

1RM Leg Press

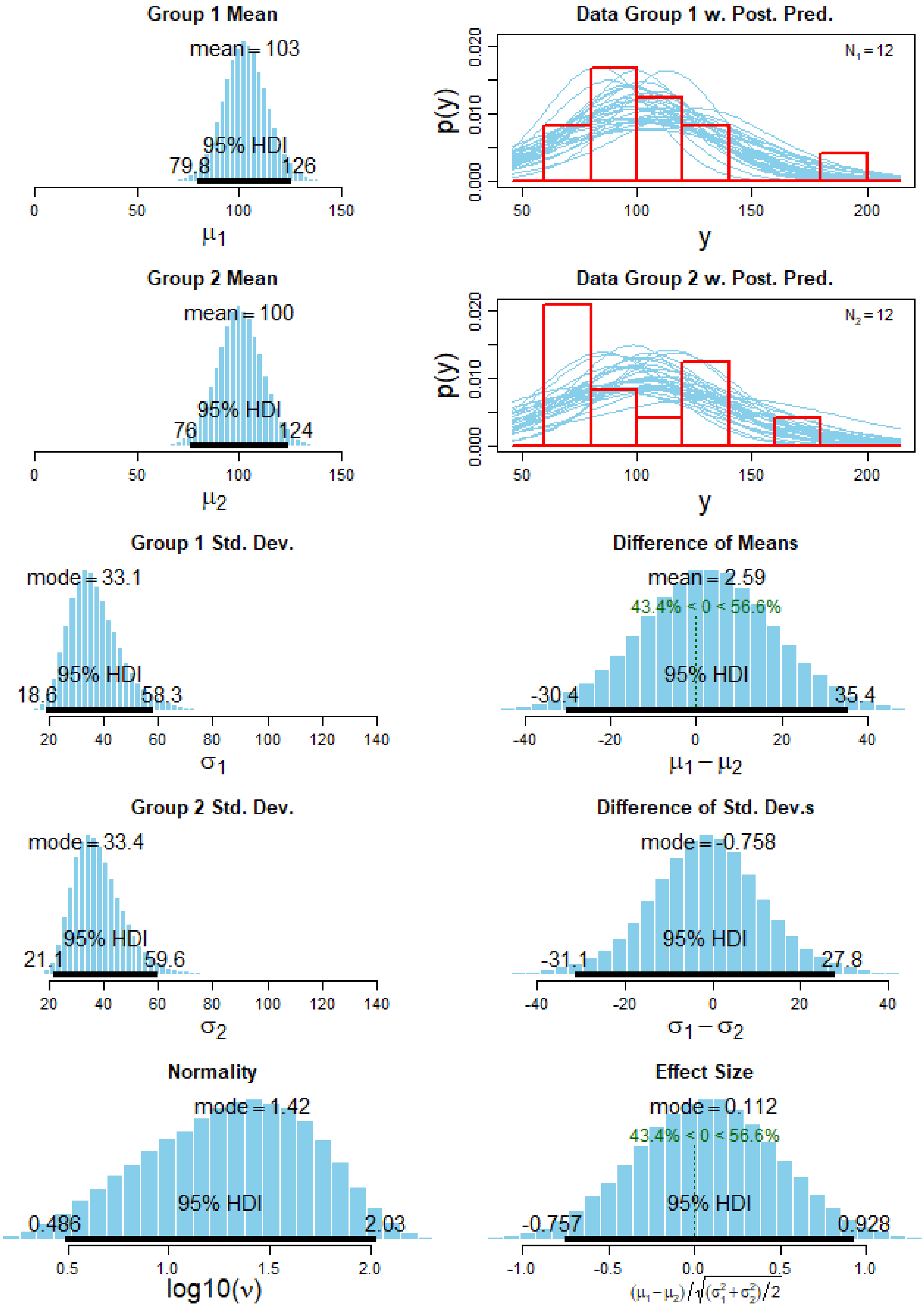

Group 1 = Test day 8

Group 2: Test day 14

1RM Leg Press

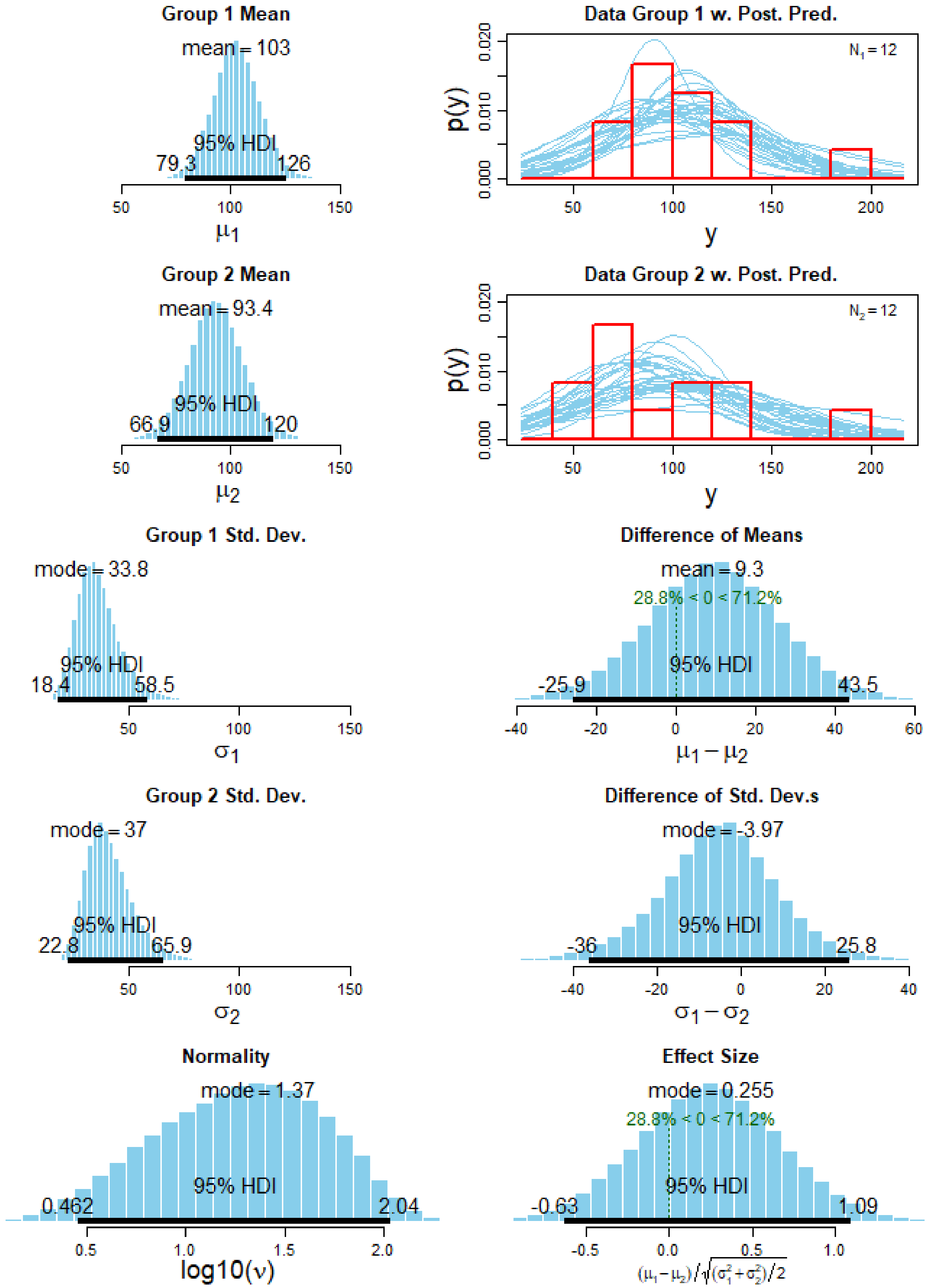

Group 1 = Test day 2

Group 2: Test day 8

1RM Leg Press

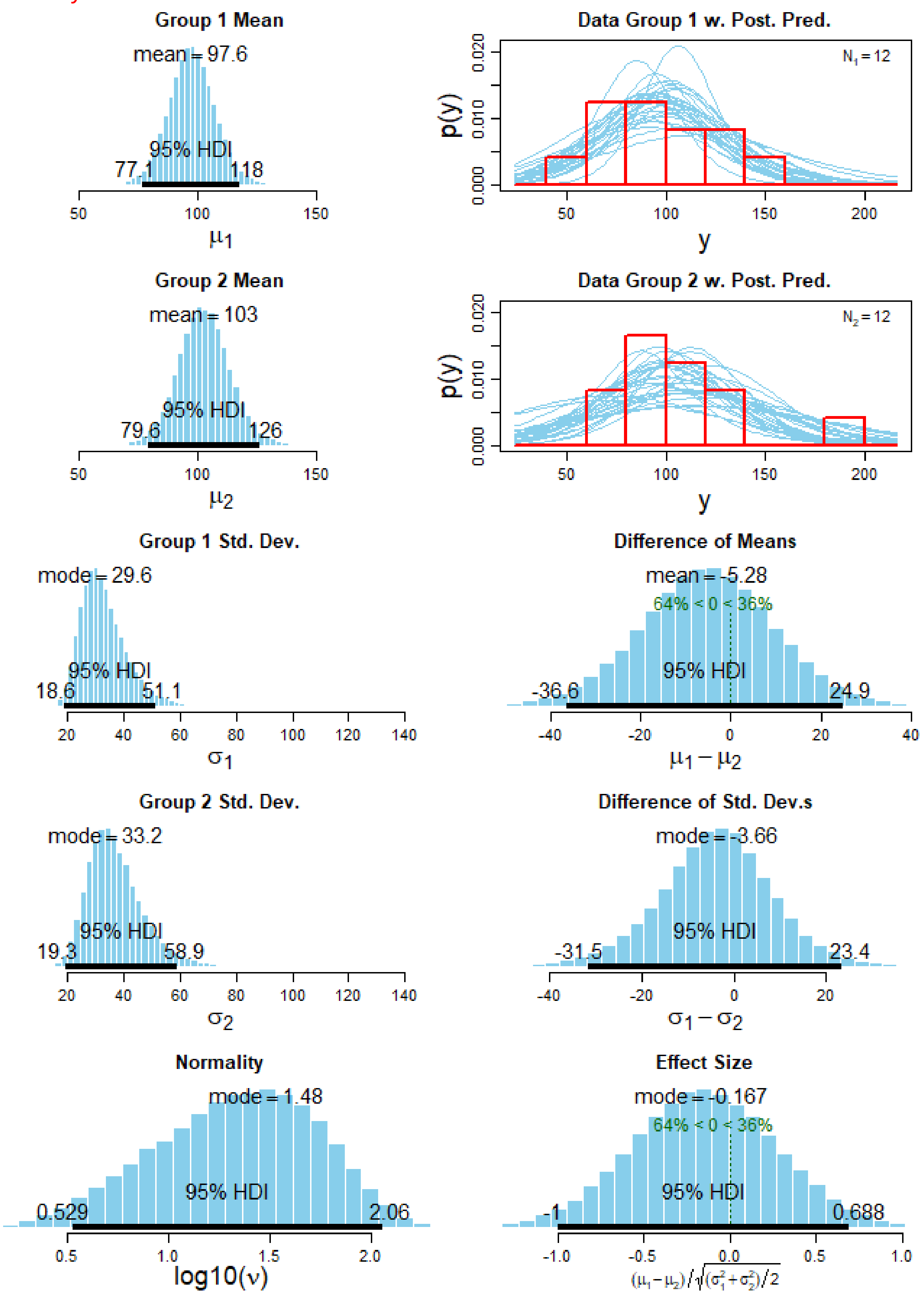

Group 1 = Test day 2

Group 2: Test day 8

1RM Leg Press

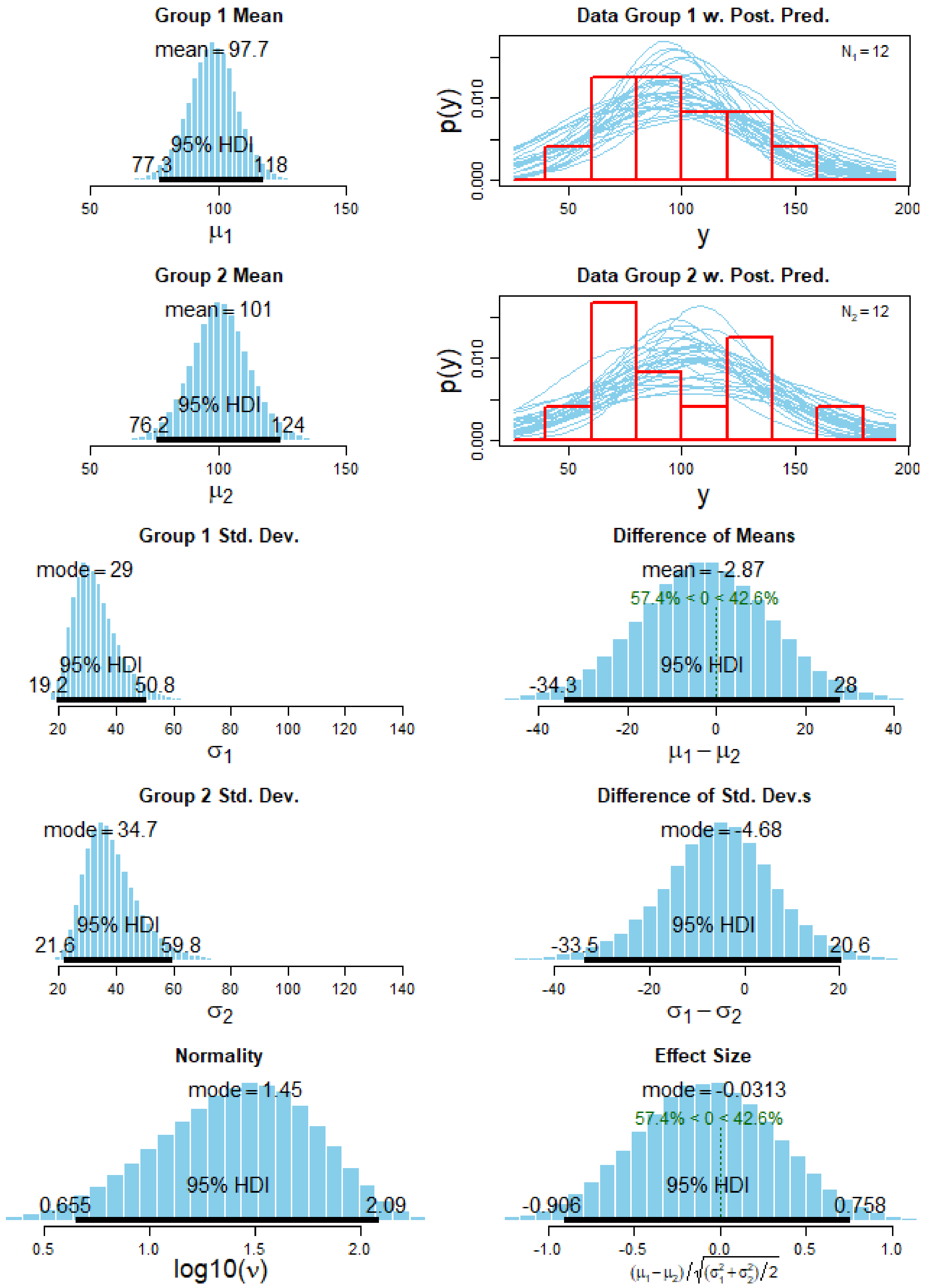

Group 1 = Test day 2

Group 2: Test day 14

1RM Leg Press

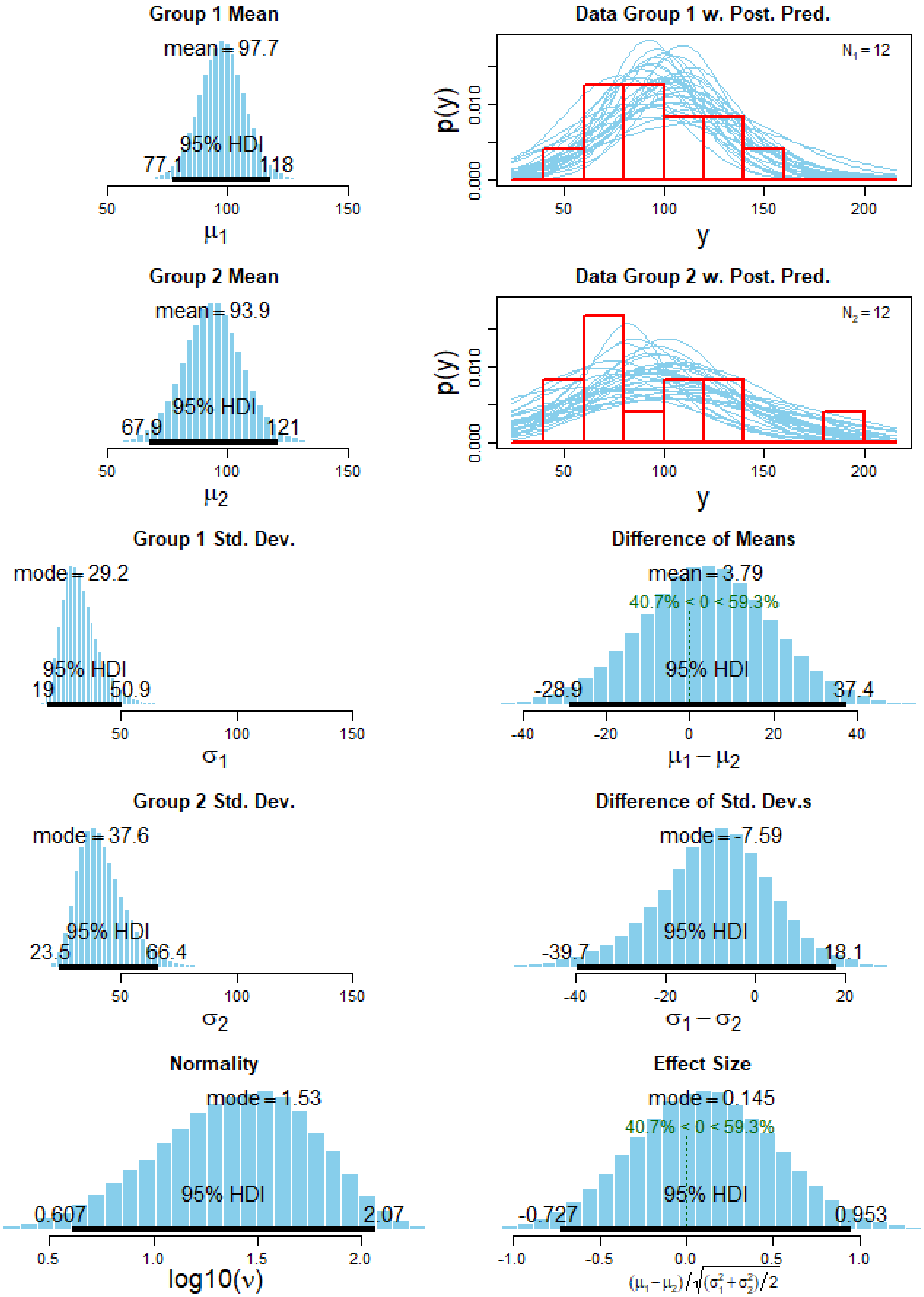

Group 1 = Test day 14
Group 2: Test day 21

1RM Leg Press

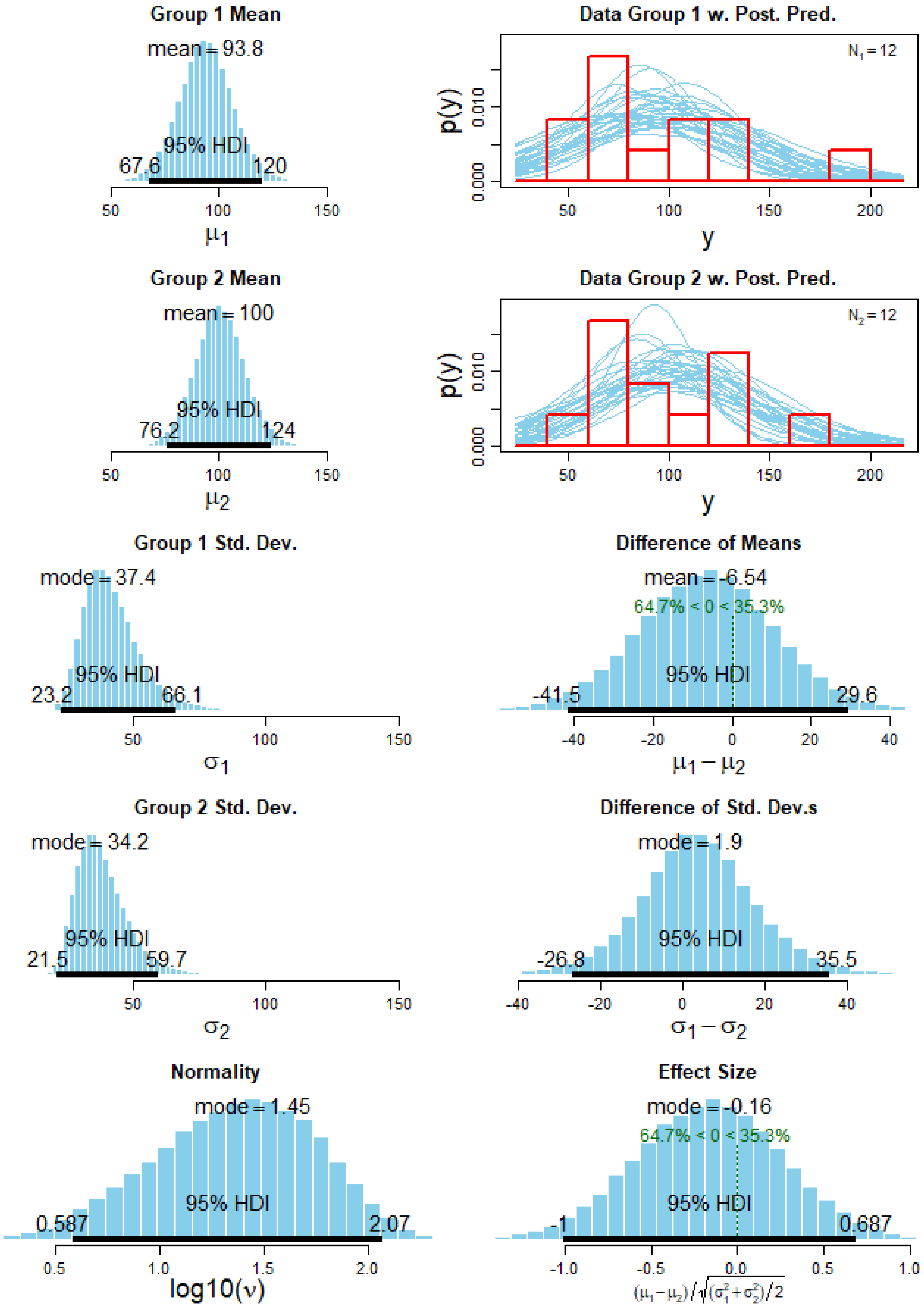

Group 1 = Test day 8

Group 2: Test day 21

Number of Push ups

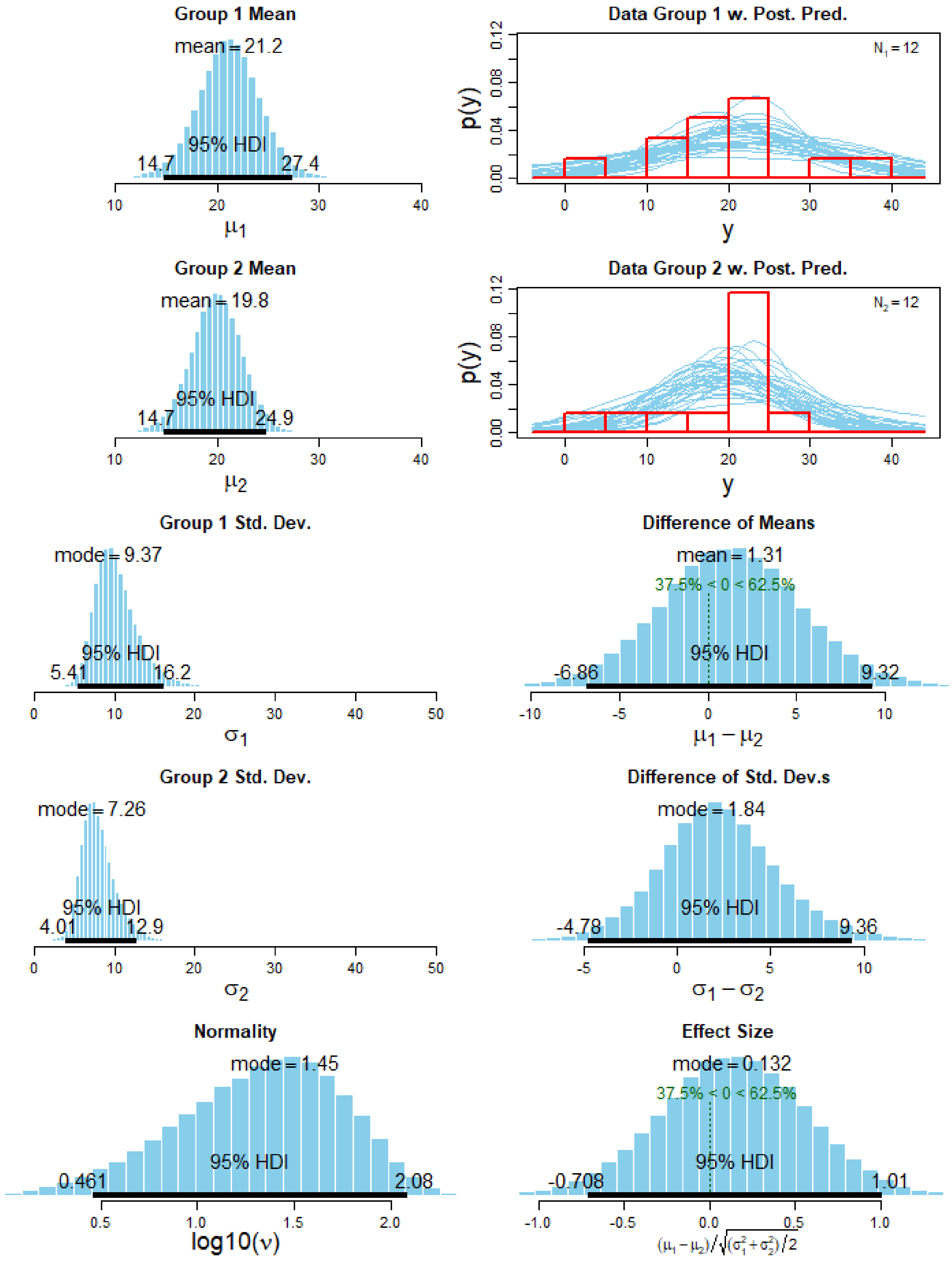

Group 1 = Test day 8
Group 2: Test day 14

Number of Push ups

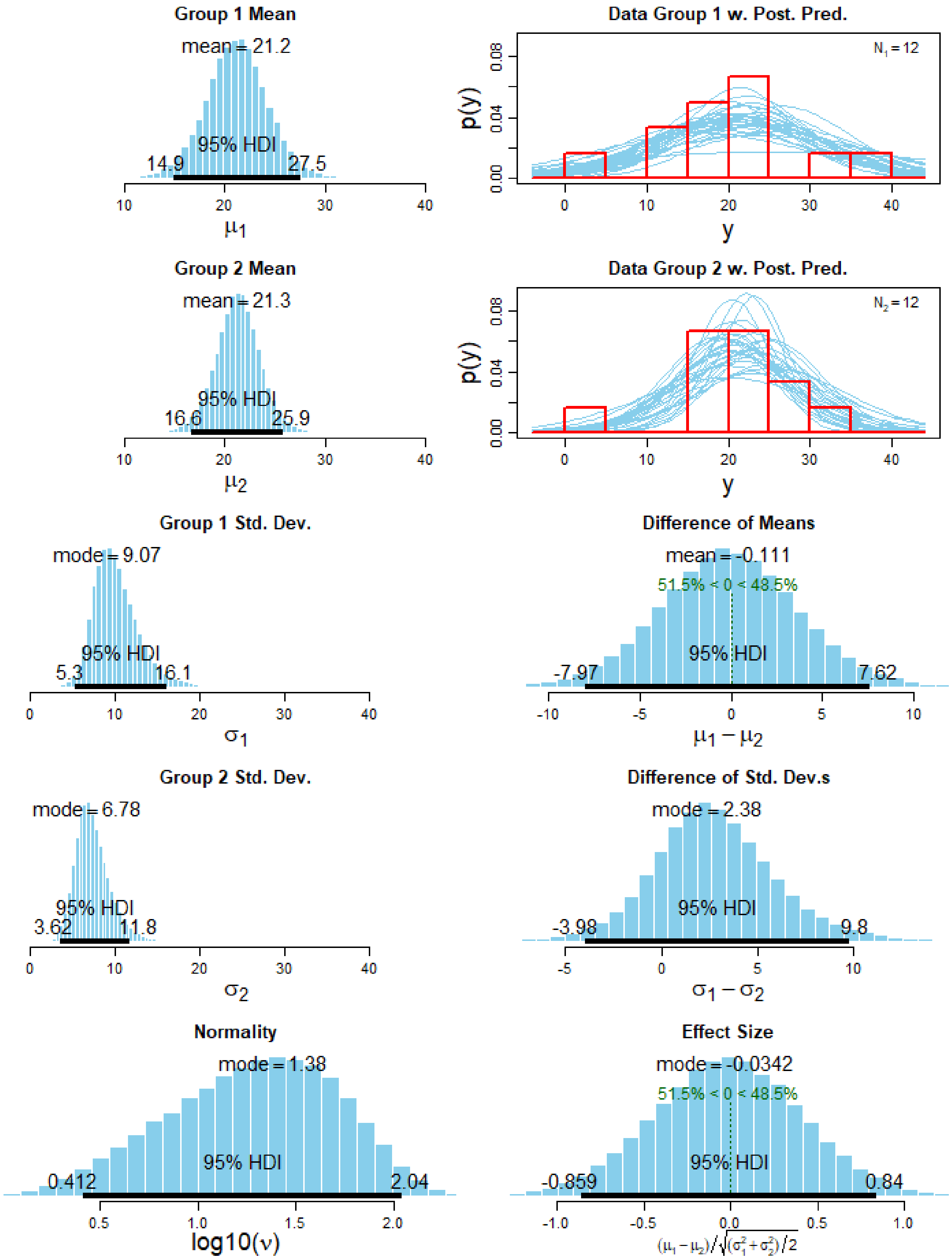

Group 1 = Test day 2

Group 2: Test day 8

Number of Push ups

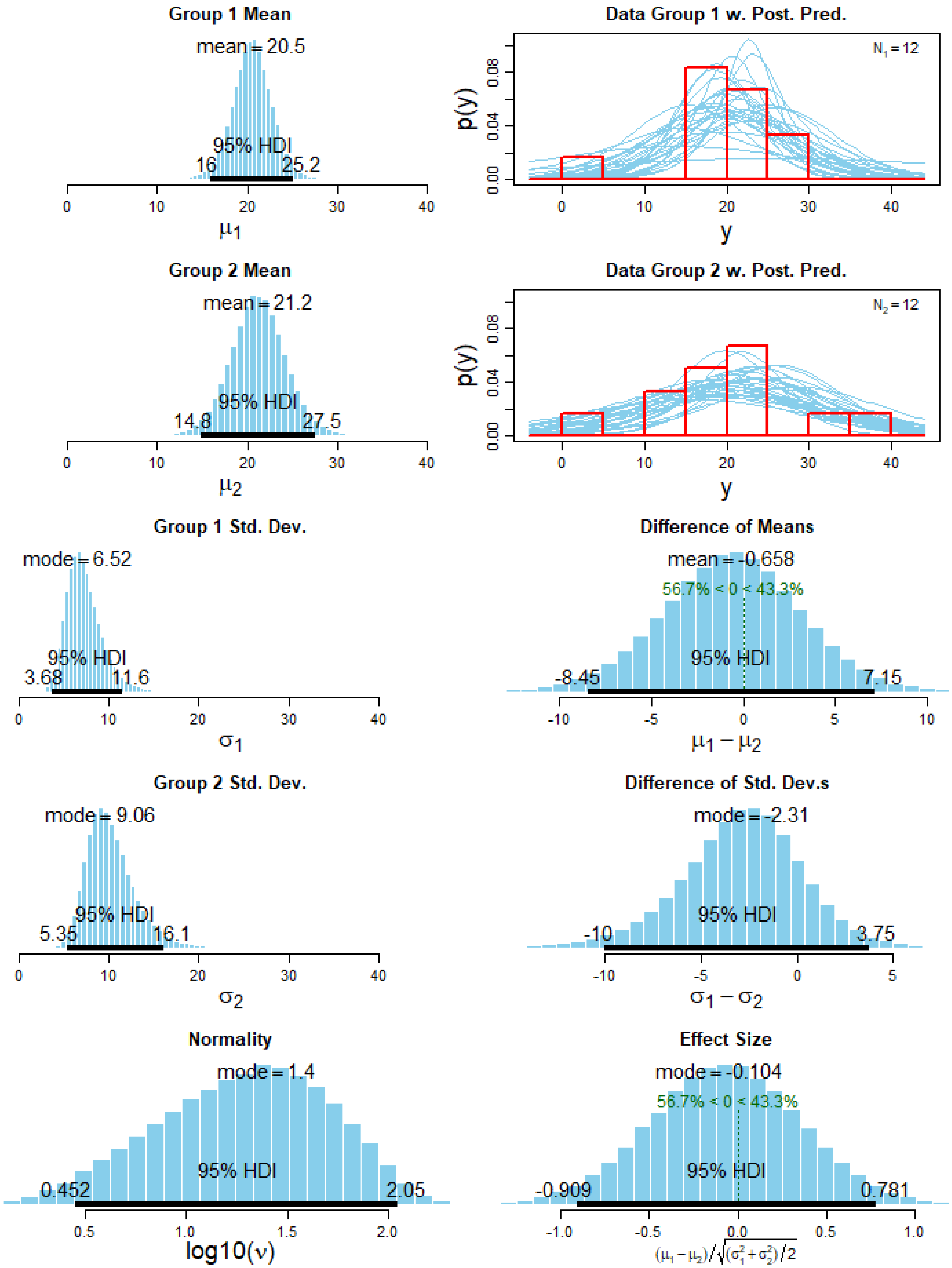

Group 1 = Test day 2

Group 2: Test day 21

Number of Push ups

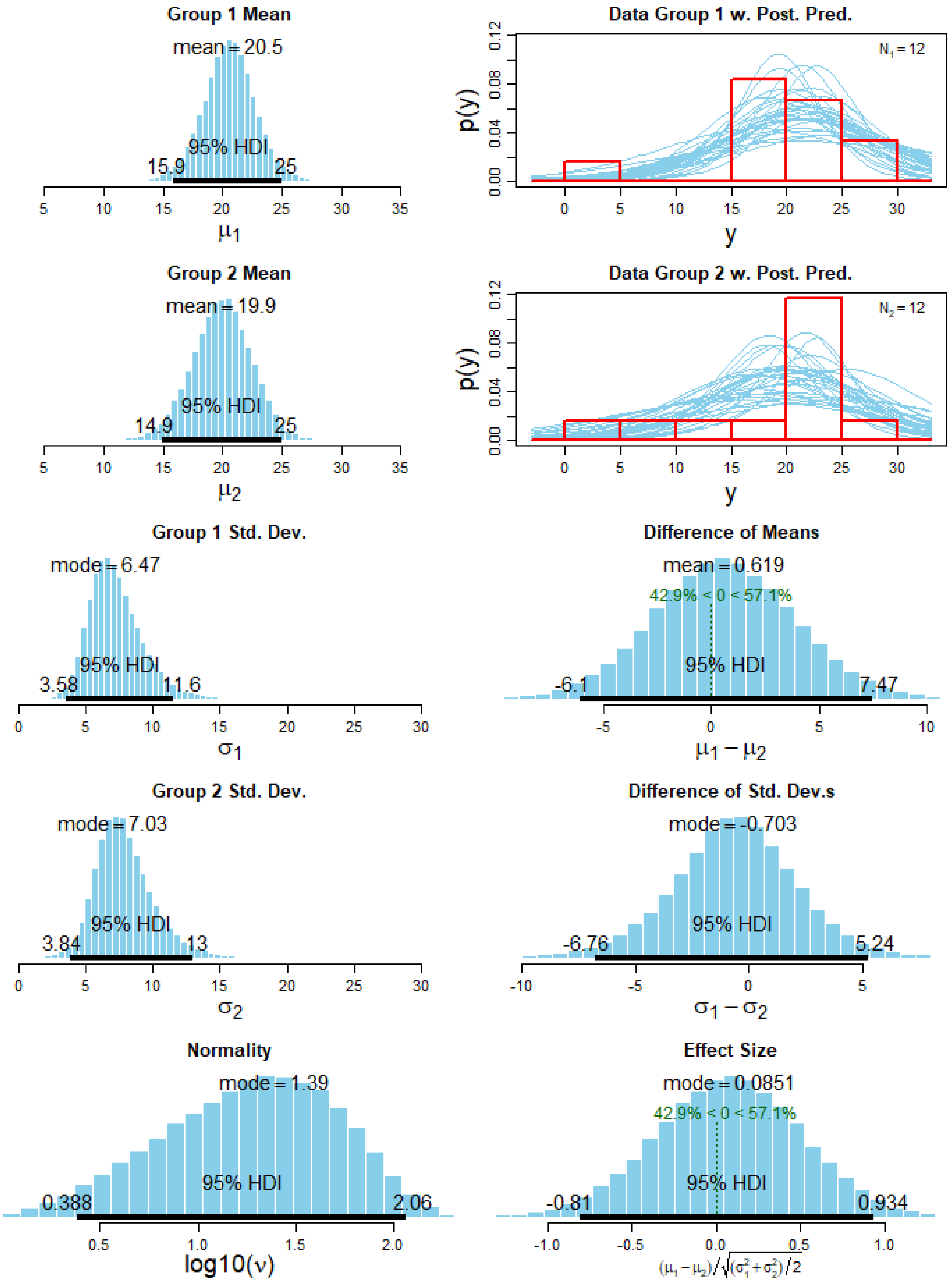

Group 1 = Test day 2

Group 2: Test day 14

Number of Push ups

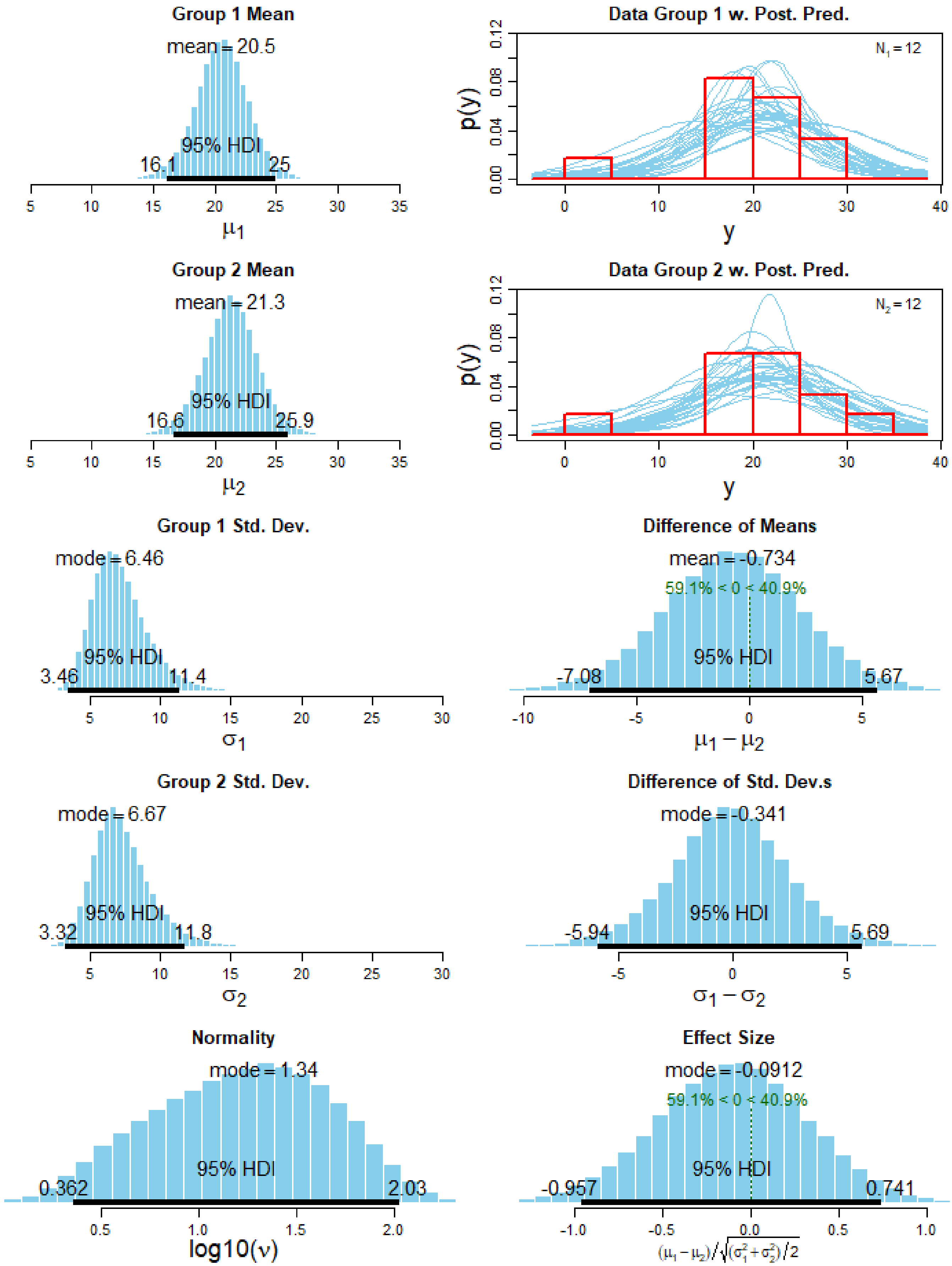

Group 1 = Test day 14

Group 2: Test day 21

Number of Push ups

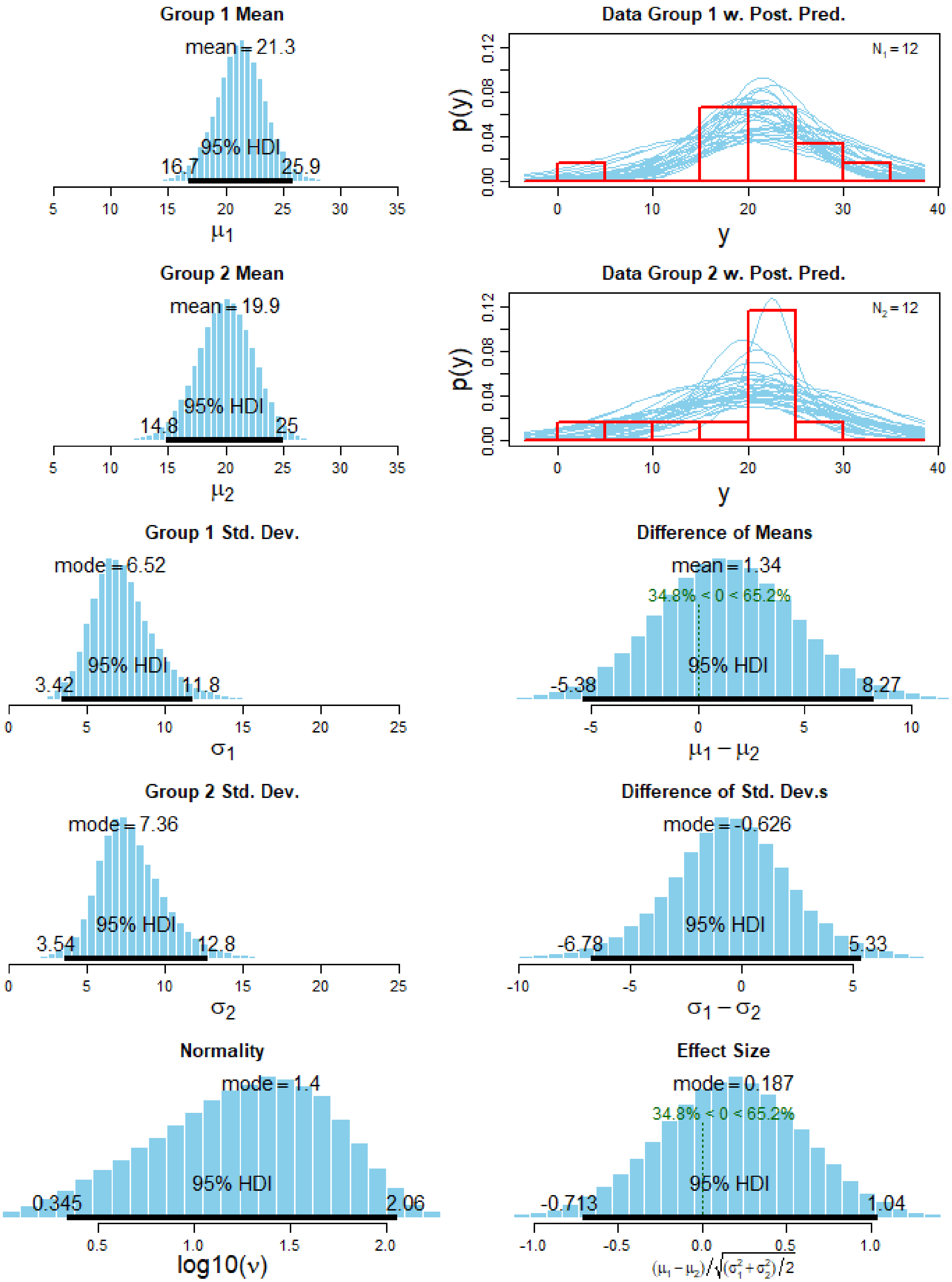

Group 1 = Test day 8

Group 2: Test day 21

Number of Leg press

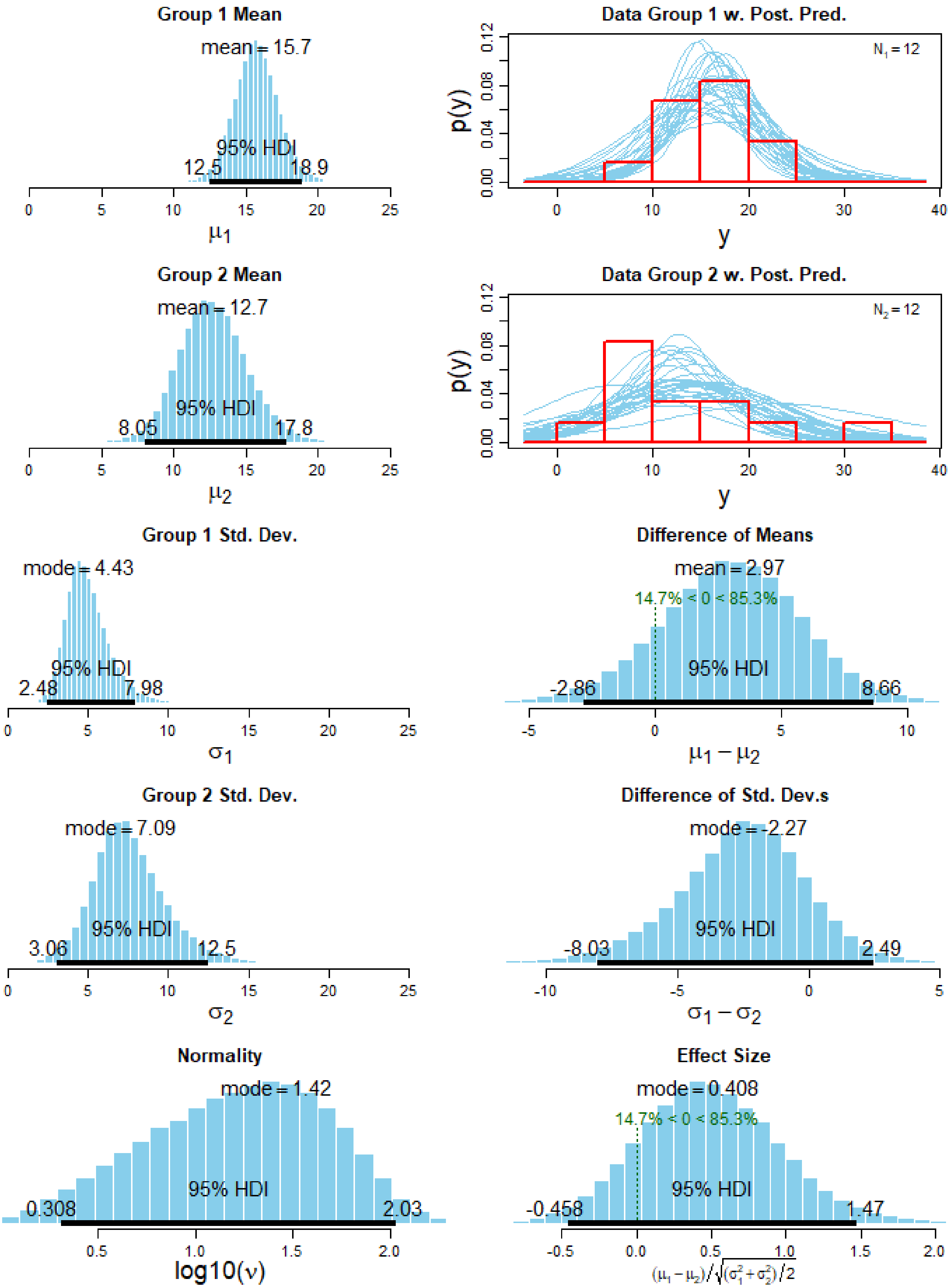

Group 1 = Test day 8
Group 2: Test day 14

Number of Leg press

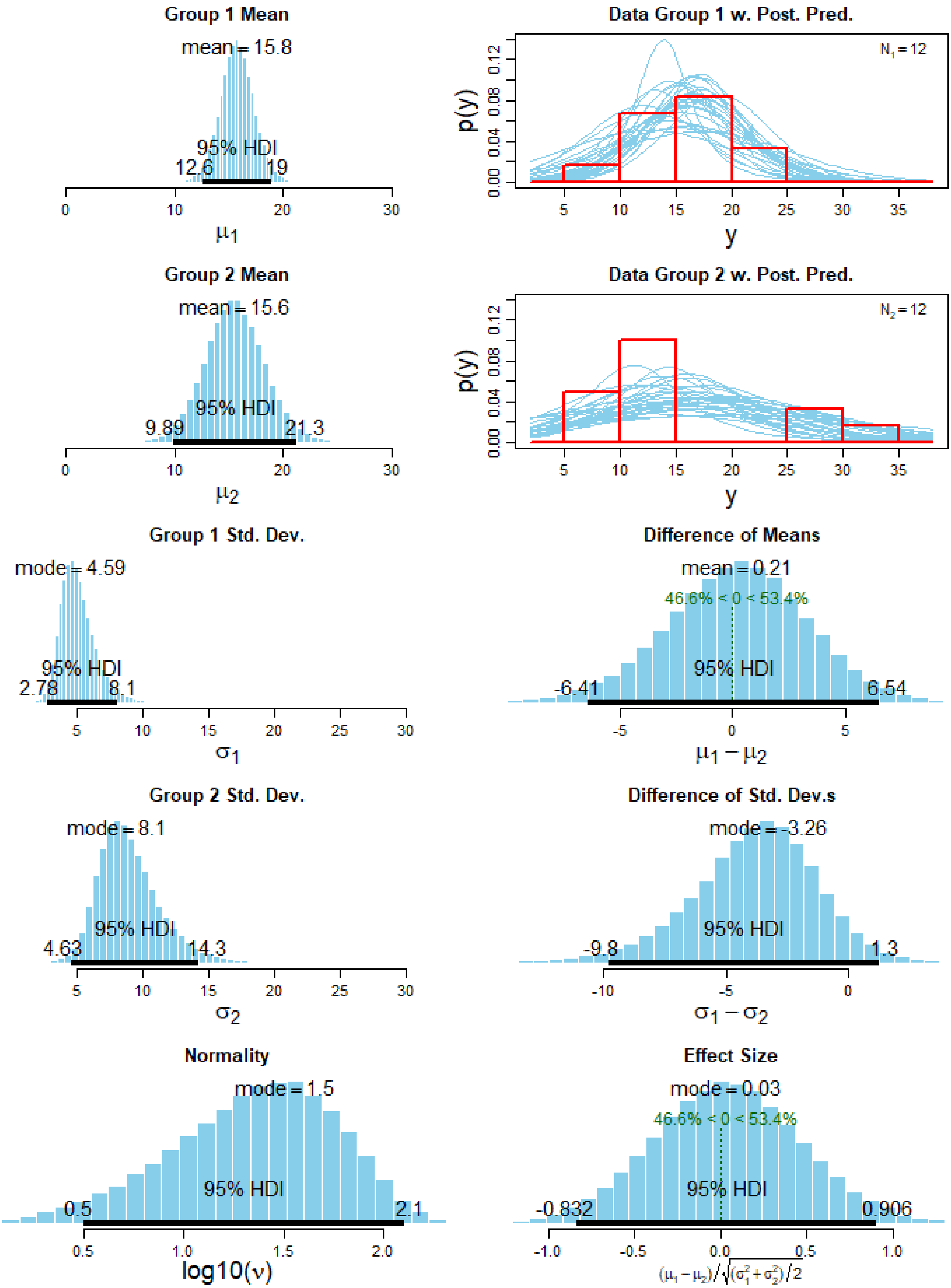

Group 1 = Test day 2
Group 2: Test day 8

Number of Leg press

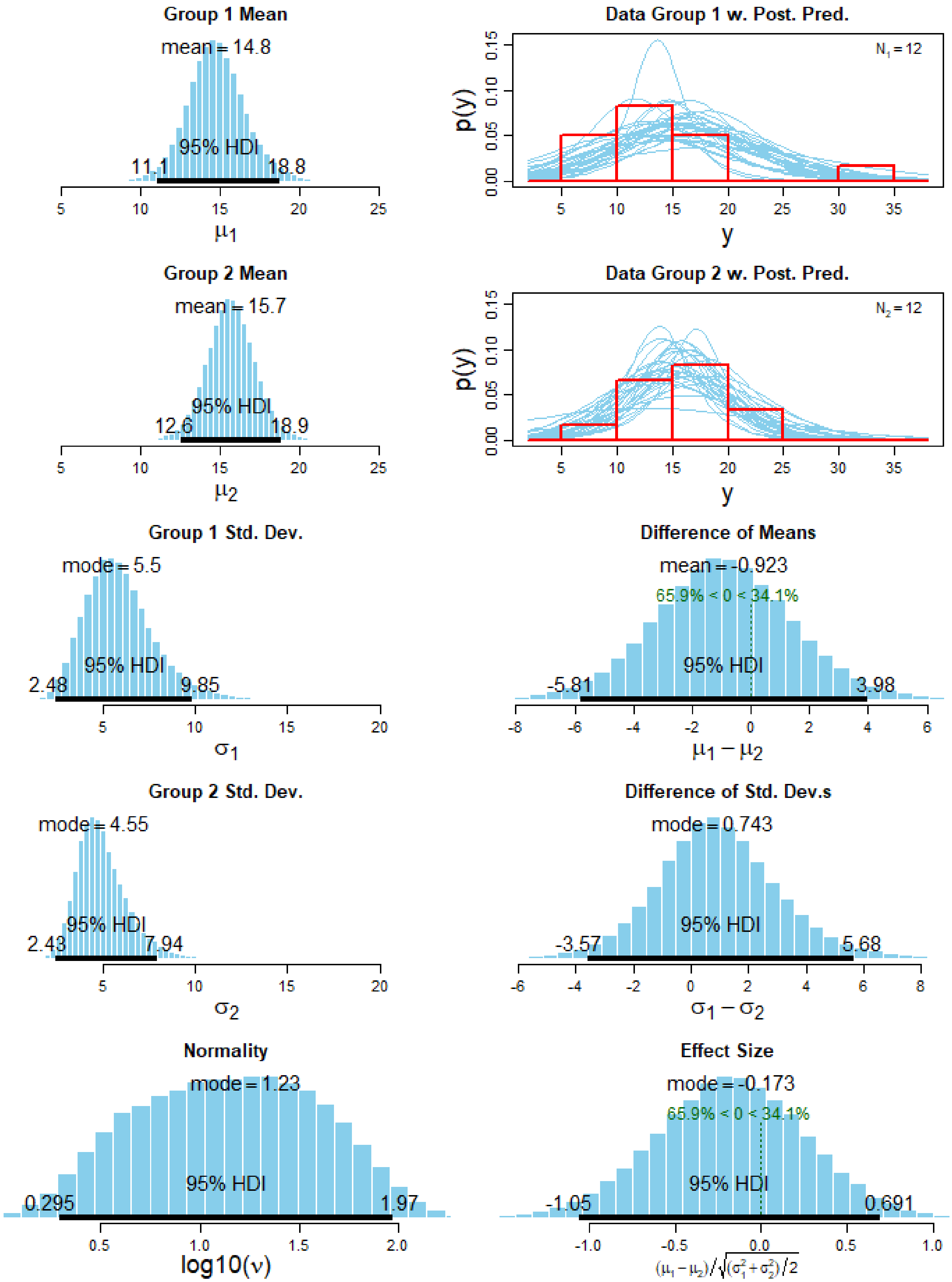

Group 1 = Test day 2

Group 2: Test day 21

Number of Leg press

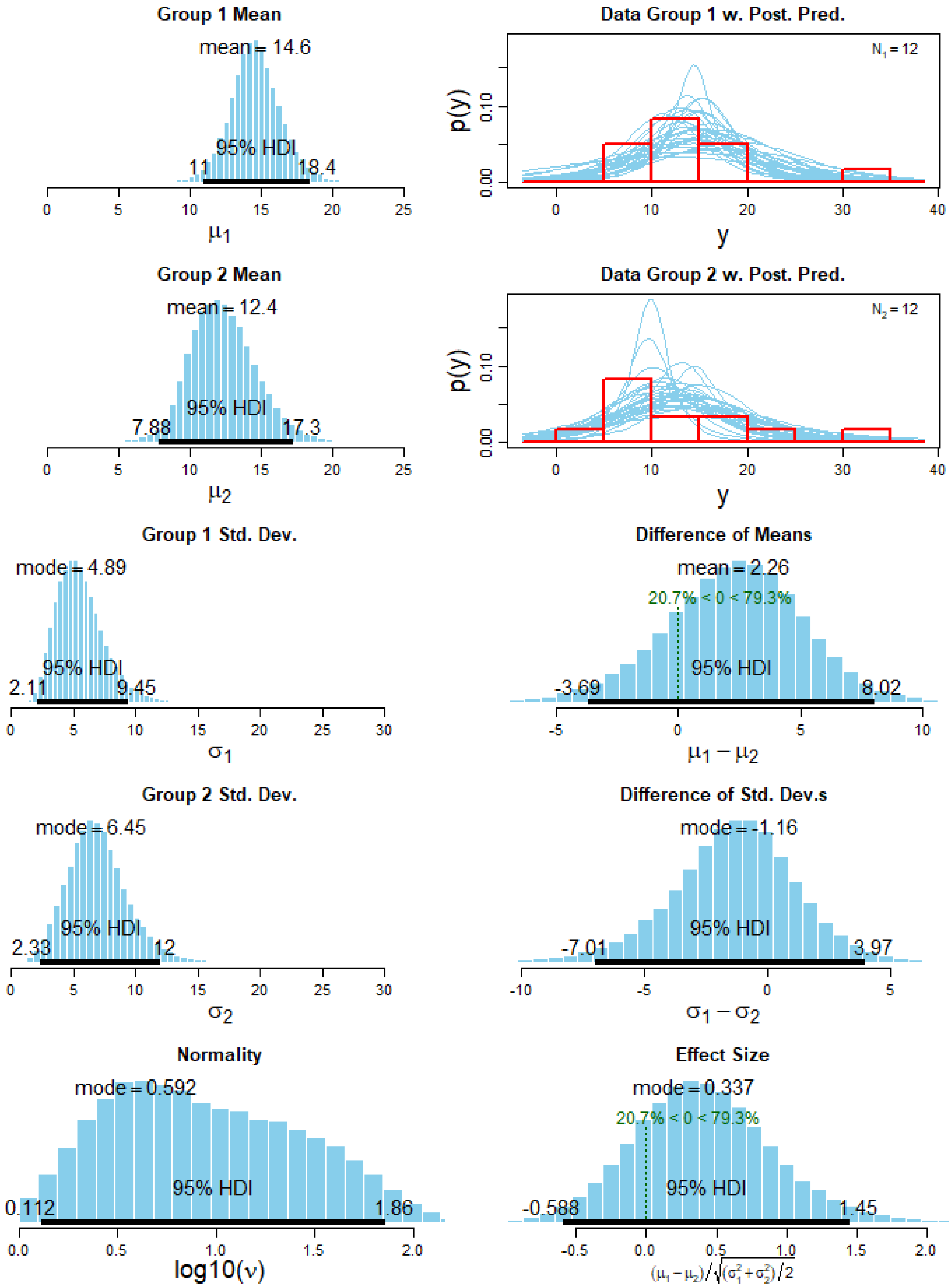

Group 1 = Test day 2

Group 2: Test day 14

Number of Leg press

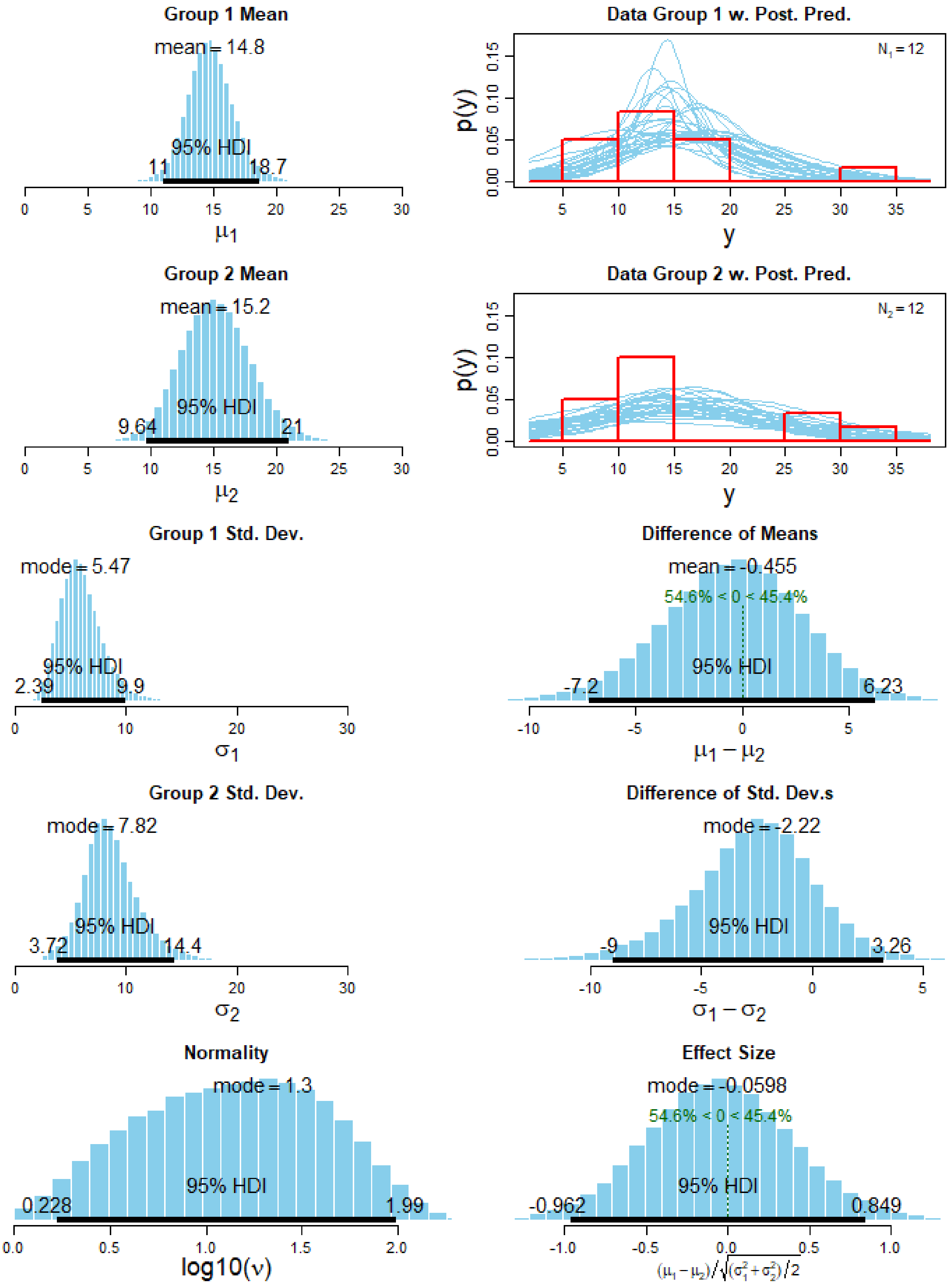

Group 1 = Test day 14

Group 2: Test day 21

Number of Leg press

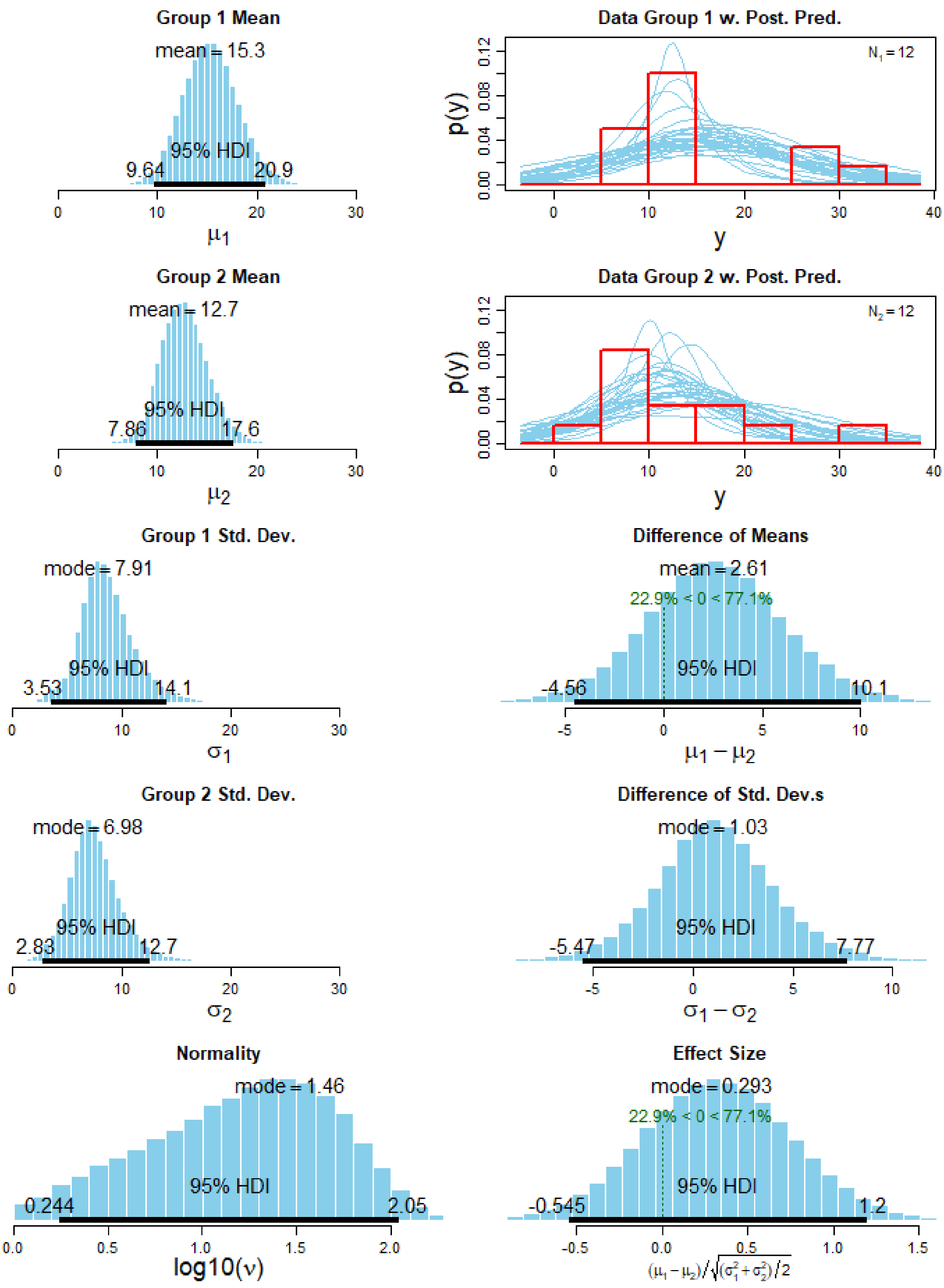

Group 1 = Test day 8

Group 2: Test day 21

Time 1600 m in seconds

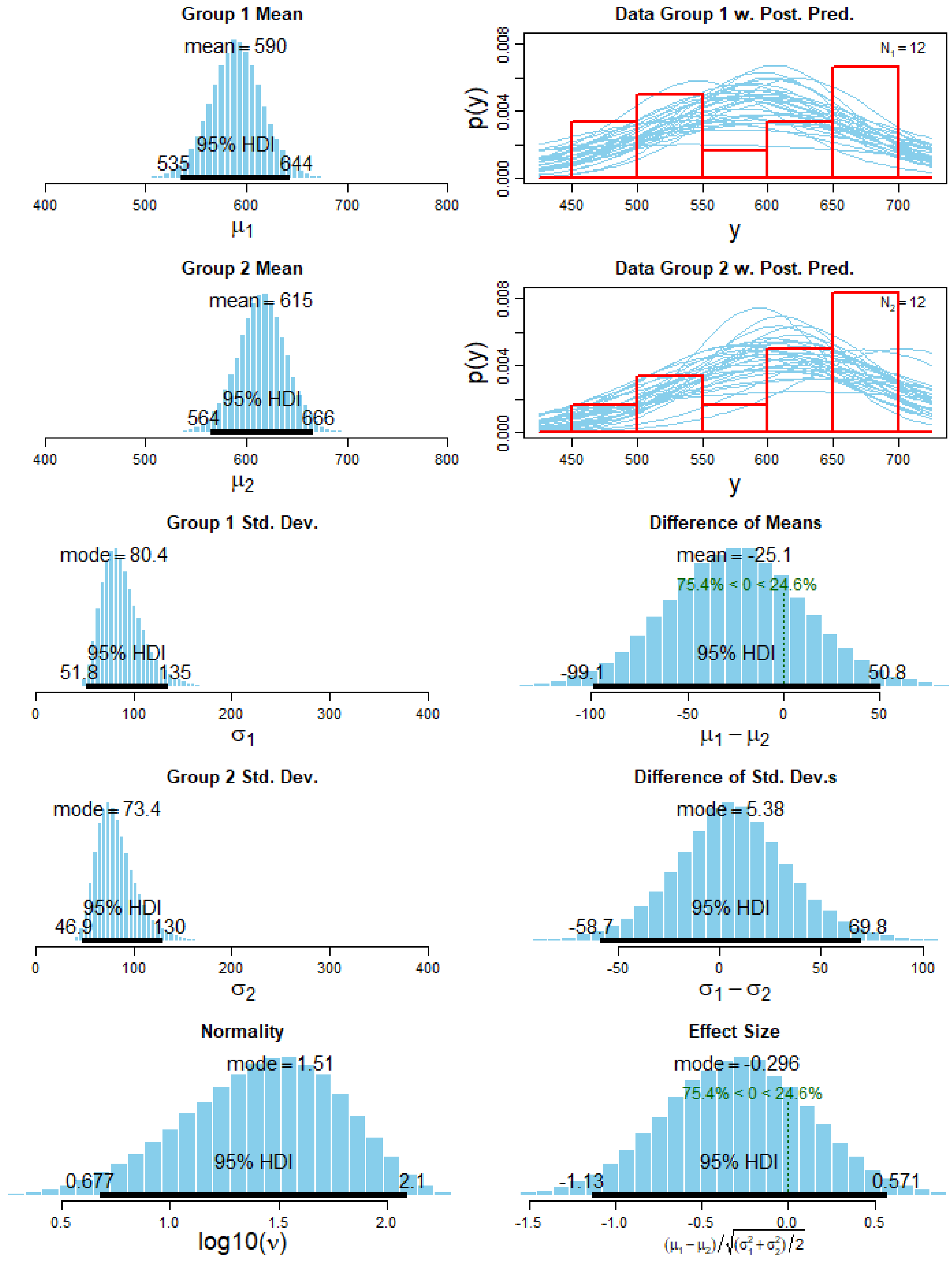

Group 1 = Test day 8

Group 2: Test day 14

Time 1600 m in seconds

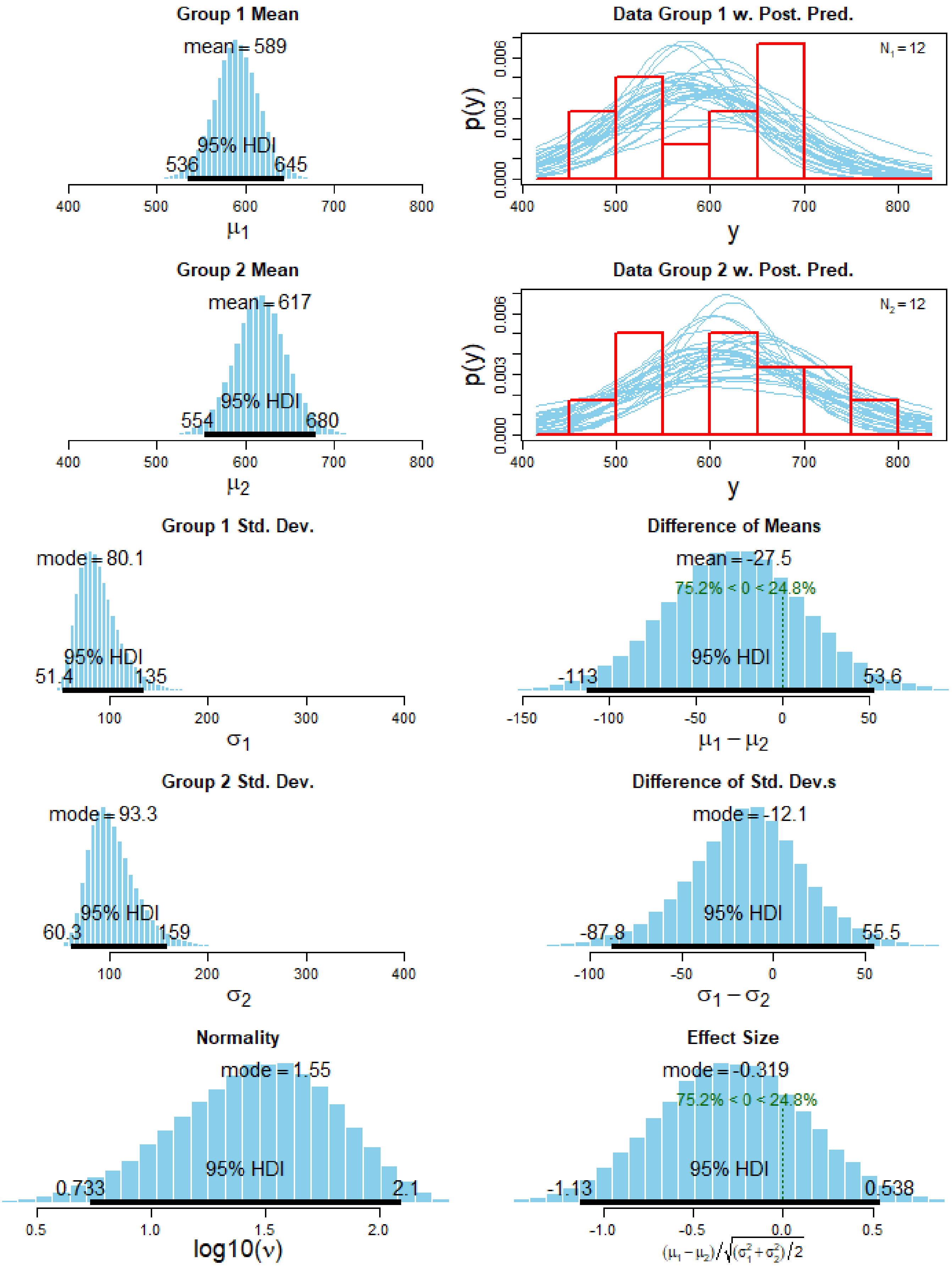

Group 1 = Test day 2

Group 2: Test day 8

Time 1600 m in seconds

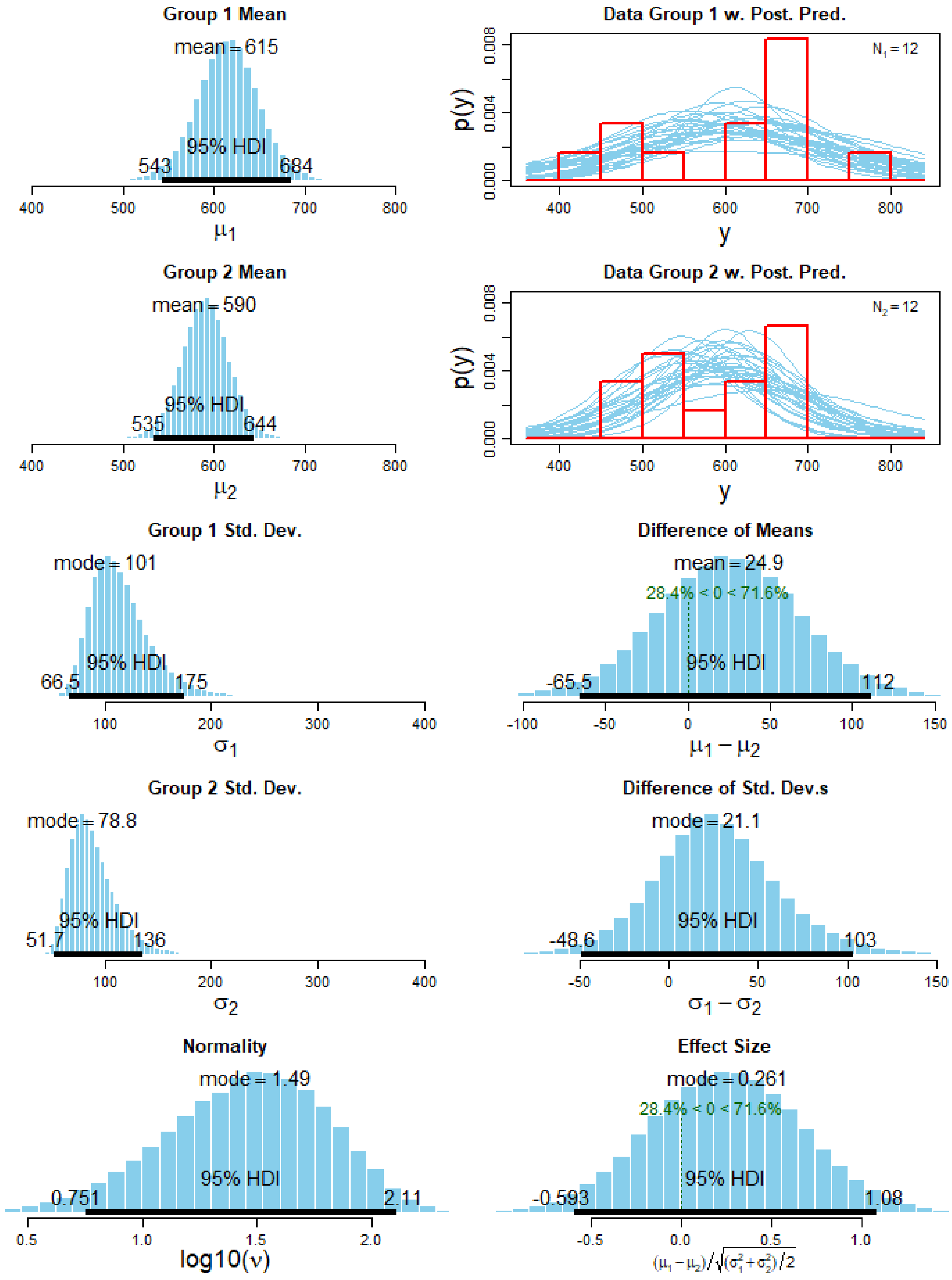

Group 1 = Test day 2

Group 2: Test day 21

Time 1600 m in seconds

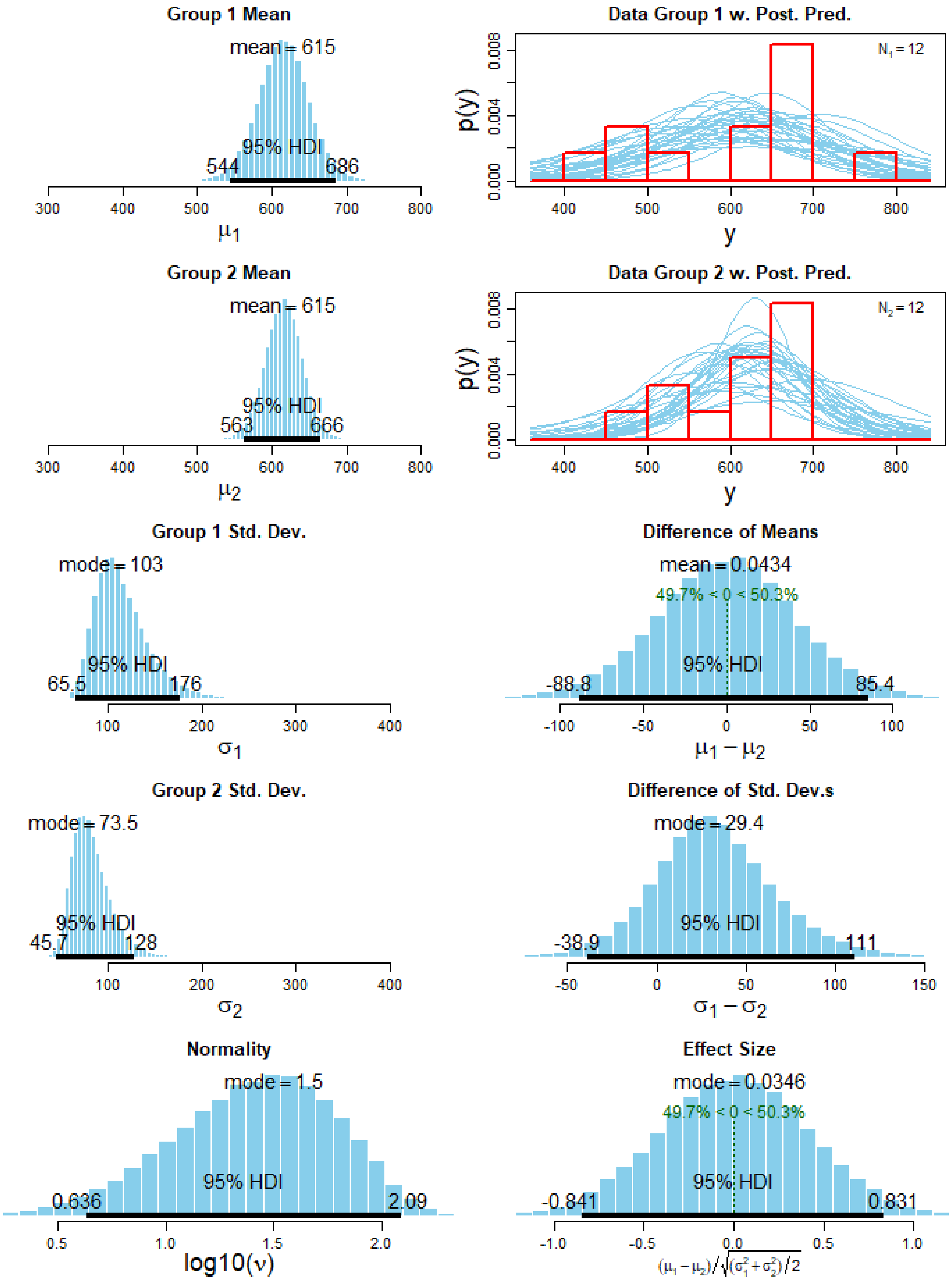

Group 1 = Test day 2

Group 2: Test day 14

Time 1600 m in seconds

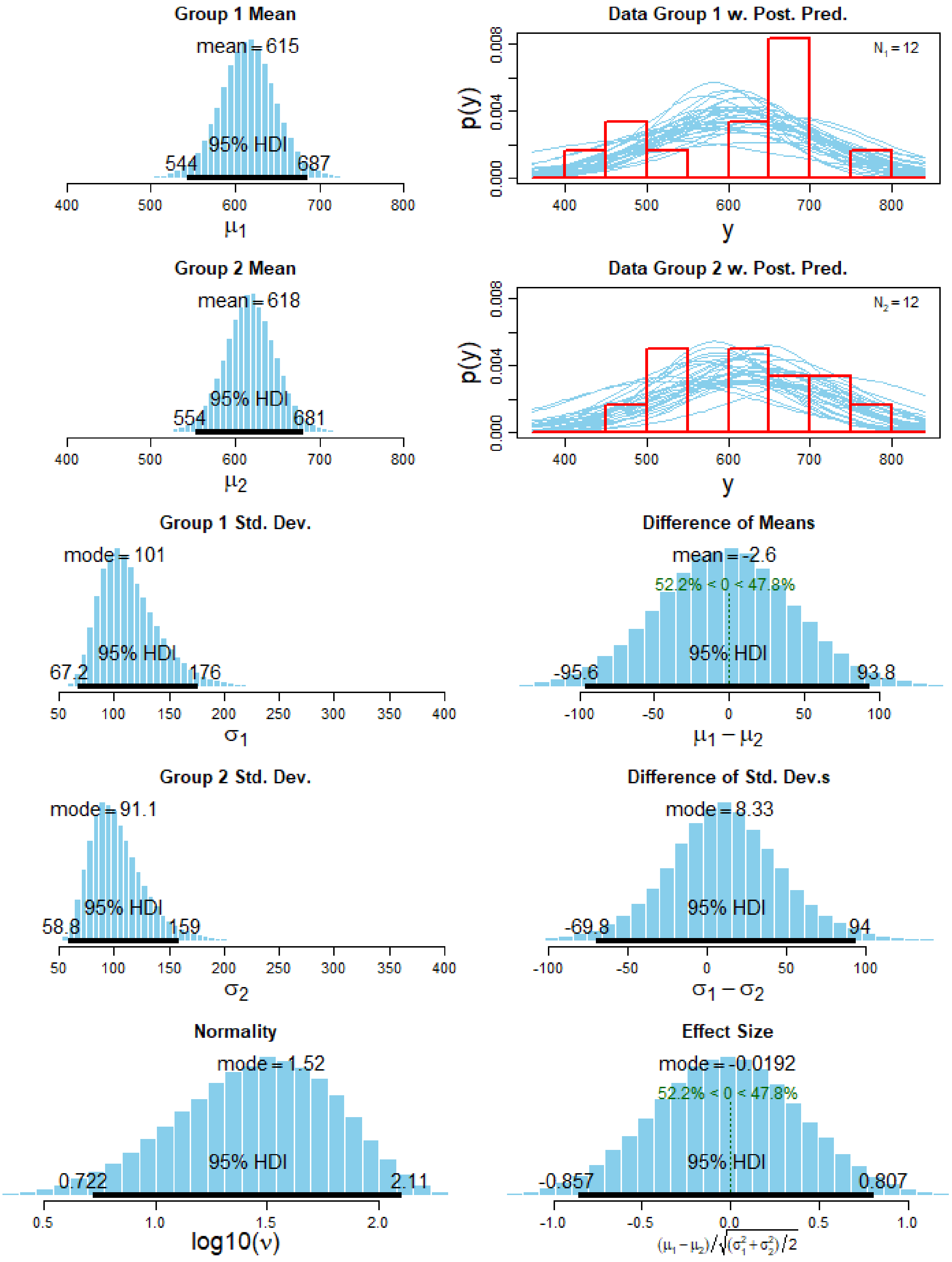

Group 1 = Test day 14

Group 2: Test day 21

Time 1600 m in seconds

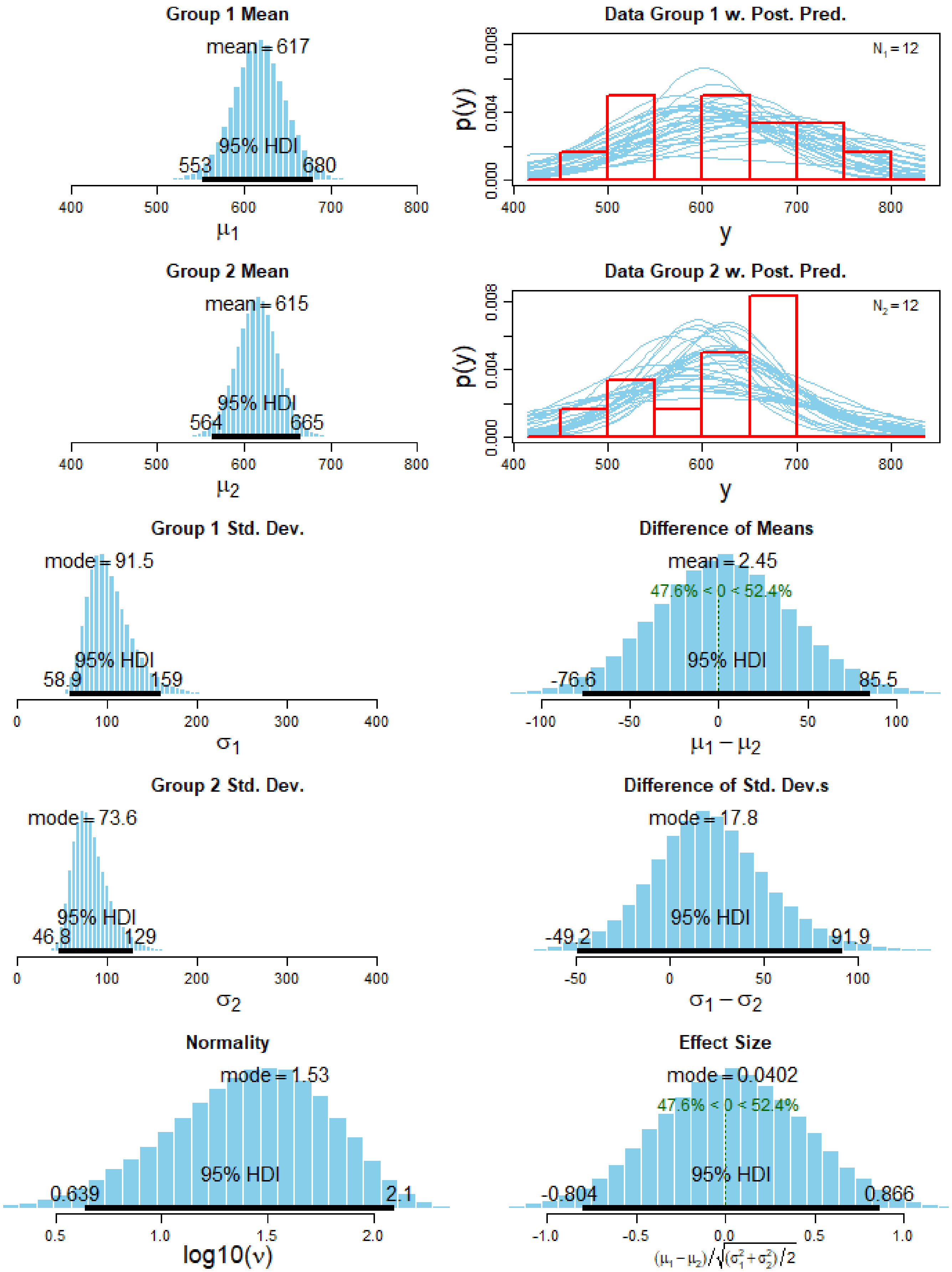

Supplement: Supplementary file 1 [file sports-09-00130-s001.zip › S1 Results and diagnostics.pdf]
